# Leucine-rich repeat kinase 2 (LRRK2): balancing cellular homeostasis and Parkinson’s disease (PD) pathogenesis

**DOI:** 10.1080/07853890.2026.2677288

**Published:** 2026-06-03

**Authors:** Iman Aolymat, Diala Walid Abu-Hassan, Aya Khleaf Oleimat, Wasan Sameer, Ban Wreikat, Tala Iqilan, Hafez Al-Momani, Heba Ali, Lubna Tahtamouni, Mahmoud Iqsairi, Mohammed Albeik, Omar Debas

**Affiliations:** a Department of Anatomy, Physiology and Biochemistry, Faculty of Medicine, The Hashemite University, Zarqa, Jordan; b Department of Physiology and Biochemistry, School of Medicine, The University of Jordan, Amman, Jordan; c Department of Microbiology, Pathology and Forensic Medicine, Faculty of Medicine, The Hashemite University, Zarqa, Jordan; d Department of Basic Dental Sciences, Faculty of Dentistry, The Hashemite University, Zarqa, Jordan; e Department of Biology and Biotechnology, Faculty of Science, The Hashemite University, Zarqa, Jordan

**Keywords:** Neurodegeneration, molecular mechanisms, diagnostic innovations, oxidative stress, therapeutic development

## Abstract

**Background:**

Leucine-rich repeat kinase 2 (LRRK2) is a kinase with multi-signalling function that regulates various processes essential for neuronal and systemic physiology. It is involved in autophagy, vesicular trafficking, mitochondrial dynamics, and immune response. Pathogenic mutations of LRRK2 can significantly interfere with these physiological pathways essential for neuronal homeostasis, inducing degeneration of dopaminergic neurons—a characteristic feature of Parkinson’s disease (PD).

**Objective:**

This review comprehensively summarizes the normal cellular functions of LRRK2 and the potential impact of its dysregulation on various physiological pathways, predisposing individuals to familial and sporadic PD. The mechanistic connections between LRRK2’s kinase hyperactivity, disturbances in vesicular trafficking and redox status, systemic and neuronal inflammation, and metabolic disorders will be thoroughly discussed.

**Results:**

Dysregulation of vesicular trafficking, mitochondrial redox balance, inflammatory pathways, and metabolism promotes α-synuclein accumulation and contributes to the degeneration of nigrostriatal dopaminergic neurons, a central pathological feature of PD. Understanding the physiological role of LRRK2 across neuronal and peripheral tissues uncovers its connection with multiple pathways to maintain homeostasis. Its dysfunction disseminates local stresses into broader neurodegenerative changes.

**Conclusion:**

LRRK2 is implicated in multiple pathways that control neuronal integrity and neurodegeneration. Therefore, therapeutic targeting of LRRK2 could potentially help in restoring physiological function and management of PD.

## Introduction

### Overview of Parkinson’s disease and underlying genetics

Parkinson’s disease (PD) is the second most common neurodegenerative disorder that affects 1–2% of the population older than 60 years and 4% older than 85 years [1]. More than 6 million active cases of PD were reported worldwide in 2016 [2], and the number of cases is estimated to rise dramatically in the next 25 years, hitting more than 25 million cases in 2050 [3]. The disease shows a cluster of symptoms like stiffness, resting tremors, rigidity, bradykinesia, and hypokinesia. Patients may also suffer from neuropsychiatric disorders such as depression, anxiety, and cognitive abnormalities. While different pathological findings are present, the most characteristic one is the presence of Lewy bodies within substantia nigra [4]. The diagnosis of PD is solely dependent on clinical manifestations. PD currently has no known cure, and the available interventions can only help in alleviating its symptoms [5]. Though originally PD was observed to sporadically occur, different studies identified familial forms of PD that may follow autosomal dominant or autosomal recessive inheritance patterns, depending on the underlying genetic mutation. Those findings suggested a close genetic connection alongside environmental risk factors [5]. A spectrum of genes was identified to play a role in PD development and prognosis with varying effects, but the most common form was leucine-rich repeat kinase 2 (LRRK2)-related mutations. Both familial and sporadic types of PD have been linked to mutations in the LRRK2 gene [6].

### Overview of Leucine-rich repeat kinase 2 (LRRK2)

**Figure 1.**
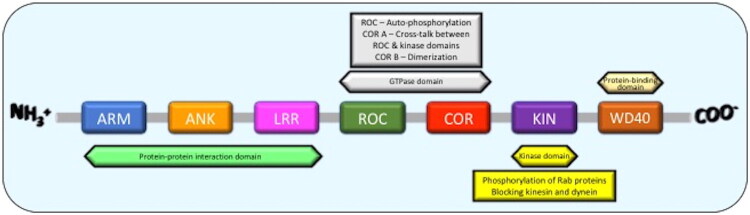
Leucine-rich repeat kinase 2 (LRRK2) protein’s domains and their functions. Diagram showing the arrangement of LRRK2 domain structure and their major function. Abbreviations: ARM, Armadillo; ANK, Ankyrin; LRR, Leucine-rich repeat; ROC, Ras of complex; COR, C-terminal of ROC; KIN, Kinase.

*LRRK2* gene is a 7449 bp gene, consisting of 51 exons and is located on chromosome 12p12, coding for a 2,527 amino acid long protein [7]. The protein encoded by LRRK2 is a 286 kDa protein, and is divided into 7 domains in the following order from the N-terminus to the C-terminus: Armadillo (ARM), Ankyrin (ANK), Leucine-rich repeat (LRR), Ras of complex (ROC), C-terminal of Roc (COR), Kinase domain (KIN), and a WD40 domain [8]. LRRK2 exists as a homodimer, connected at the ROC-COR domains [8,9]. Functionally, LRRK2 is divided into two halves: The N-terminal domains (ARM, ANK and LRR) and the C-terminal domains (the catalytic half), consisting of ROC, COR, KIN and WD40. The domains of LRRK2 have distinct roles to assist in its overall cellular function. The N-terminal domains are mainly involved in protein-protein interactions and regulation of the catalytic functions, while the C-terminal domains have two catalytic functions, which are: serine-threonine kinase and GTPase [9]. The N-terminus domains ARM, ANK and LRR interact with cellular proteins, such as β-actin, α-tubulin, and synapsin I [10]. Also, the armadillo domain facilitates LRRK2 and Fas-associated protein with death domain (FADD) interactions, which promotes apoptosis [10]. The ROC domain contains a GTPase enzyme which regulates LRRK2’s function *via* auto-phosphorylation. GTP hydrolysis, mediated *via* GTP-ase activity, enhances the kinase activity of LRRK2, possibly by stabilising it [11]. The COR domain is divided into COR A and COR B. COR B plays a role in the dimerization of LRRK2, whereas COR A is involved in crosstalk between the ROC and kinase domains [8]. The kinase domain is responsible for phosphorylating Rab (Ras related in brain) proteins, such as Rab8, Rab10, and Rab29 [12]. Phosphorylation of Rab GTPases by LRRK2 regulates endolysosomal vesicle trafficking, including cargo delivery to lysosomes and lysosomal positioning and maturation, and an increase in Rab phosphorylation plays a role in PD pathogenesis [12]. More importantly, it is also in the kinase domain that the G2019S mutation, which is the most common genetic determinant of PD, occurs [8]. The WD40 domain is involved in cell signalling. WD40 forms β-propeller structures which function as a platform for other proteins to interact on, such as protein assembly [13]. After the WD40 domain, a 28 – amino acid chain at the C-terminus may be involved in regulating the kinase activity [8]. Figure 1 presents the LRRK2 protein’s domains and their major functions.

## Physiological functions of LRRK2

The intracellular trafficking pathways consisting of autophagy, endocytosis, the trans-Golgi network (TGN), and mitochondrial quality control, constitute a densely integrated network that maintains homeostasis in living creatures. Recently, the LRRK2 protein has been recognized as a key driver at the intersection of these networks, with pathogenic LRRK2 variances being increasingly linked with PD development.

## Physiological role of LRRK2 in autophagy

Autophagy is a conserved catabolic cellular process that destroys improperly folded proteins, malfunctioning organelles, and infectious agents through lysosomal degradation. Autophagy starts with the encapsulation of cellular cargo *via* double-membraned autophagosomes which transport the cargo and fuse it with lysosomes. Several previous studies have shown that LRRK2 has been actively involved in various steps of this system. For instance, LRRK2 regulates acidification of lysosomes and lysosome-autophagosome fusion *via* its interaction with the vacuolar-type H+-ATPase pump a1 subunit. This interaction is compromised by LRRK2 mutations, resulting in impaired lysosomal-mediated destruction of waste products [14]. Several investigations have shown that LRRK2 kinase activity influences essential autophagy modulators, including Rab GTPases, which are proteins able to bind to cellular membranes *via* the C-terminal. Their functions vary regarding intracellular trafficking, from cellular secretory pathways to intracellular degradation pathways of lysosomal system, through more than one Rab GTPase [15]. LRRK2 can phosphorylate several members of the Rab family of small GTPases [16,17], such as Rab1, Rab3, Rab5, Rab8, Rab10, Rab12, Rab29, Rab35, and Rab43. Of these, Rab29 plays a crucial role in recruiting LRRK2 to the trans-Golgi network and thereby the activation of its kinase activity [18,19]. Activated LRRK2 subsequently phosphorylates Rab substrates such as Rab8, Rab10, and Rab12, which participate in vesicular trafficking regulation [20]. Stressed lysosomes recruit LRRK2 *via* Rab29, resulting in activation of LRRK2 and stabilization of Rab8/Rab10 by phosphorylation, protecting against lysosomal disruption. This protective effect of LRRK2 is mediated *via* Rab effectors, including EHBP1/EHBP1L1 which enhance secretion [21]. Moreover, LRRK2 mutations result in mislocalization of Rab8a, which is a fundamental regulator of endocytic recycling. This is associated with disrupted transferrin trafficking and intracellular iron accumulation [22]. G2019S and R1441C mutations of LRRK2 are linked to impaired autophagy and dysregulated lysosomal structure and function [23,24]. Recently, the role of LRRK2 in formation of autophagosome and phagophore, fusion of autophagosome and lysosome, maturation of lysosomes, destruction of lysosomal protein, and regulation of lysosomal pH and calcium homeostasis has been identified [23].

In addition to its role in canonical autophagy, LRRK2 is also involved in noncanonical autophagy pathways, such as conjugation of ATG8 to single membranes (CASM). CASM involves the lipidation of ATG8 family proteins on single-membrane compartments of the endolysosomal system, which facilitates the recruitment and activation of LRRK2 at stressed lysosomes and phagosomes. This interaction links LRRK2 signalling to lysosomal stress responses and membrane remodelling [25]. Furthermore, stimulator of interferon genes (STING) pathway is associated with CASM-mediated lipidation of ATG8 proteins activation, particularly GABARAPs, which promotes LRRK2 recruitment to lysosomes and enhances its kinase activity [26]. Consistent with these findings, non-canonical autophagy or CASM activates LRRK2 through ATG8 lipidation, contributing to the regulation of endolysosomal homeostasis and lysosome-associated signalling pathways [27]. Collectively, these studies highlight CASM-dependent pathways as important regulators of LRRK2 activity in lysosomal stress responses.

The evolving role of LRRK2 in chaperone-mediated autophagy (CMA), including destruction of cytosolic proteins containing the KFERQ motif has been established. G2019S mutation inhibits LAMP2A (a crucial lysosomal membrane protein that acts as the specific receptor and translocation channel for CMA) multimerization which is required for substrate movement across lysosomal membrane [28]. Consequently, impaired CMA and autophagy is associated with accumulation of toxic products, such as α-synuclein, play critical role in PD pathogenesis. In addition, patients carrying LRRK2 variant p.G2294R in the WD40 domain have shown compromised capacity of macrophage to clear pathogens and α-synuclein fibrils [29]. Furthermore, autophagy that selectively clears damaged mitochondria (mitophagy) can be compromised by LRRK2 abnormalities, resulting in disturbances in mitochondrial homeostasis [30–32]. Therefore, LRRK2-linked abnormalities that converge in autophagy and mitochondrial quality control represents an increasingly important pathological connection.

## Physiological role of LRRK2 in endocytosis and trans-Golgi network

Endocytosis is a critical physiological function involved in importing constituents of plasma membrane and extracellular materials. Endocytosis is strictly controlled by LRRK2, which regulates vesicle fission, transport, and maturation. The role of LRRK2 in endocytosis is mediated *via* phosphorylation of endocytic Rab proteins, such as Rab5, Rab8a, Rab10, and Rab29, modulating their activity and location [18,33–35]. Particularly, Rab29 attracts LRRK2 to the trans-Golgi network and lysosomes, where it activates its kinase activity. Clathrin-mediated endocytosis (CME), a mechanism that recycles synaptic vesicles in neurons has been found to be affected by LRRK2 mutations [36,37]. Defects in this mechanism within dopaminergic neurons is associated with synaptic dysfunction and poor neurotransmission, contributing to PD. LRRK2 is also linked to endocytosis of synaptic vesicles *via* associations with dynamin and endophilin A [34]. These observations couple the activity of LRRK2 to synaptic integrity. Interestingly, endocytic vesicle trafficking intersects with autophagy at the late endosome and lysosomal level. Amphisomes created by the fusion of autophagosomes and endosomes, need coordinated vesicle trafficking that is regulated by Rab GTPases, many of which are LRRK2 substrates [38]. As a result, disruption of endocytosis mediated by LRRK2 is expected to cause abnormal synaptic transmission as well as faulty autophagy, increasing neuronal susceptibility in PD.

The trans-Golgi Network (TGN) is a sorting centre in the secretory system, guiding cargo proteins to post-Golgi compartments such as the plasma membrane, endosomes, lysosomes, and autophagic organelles. It has been established that LRRK2 is actively interacting with several proteins and pathways that control the activity of the Golgi, preserving cellular homeostasis. The TGN is highly enriched with LRRK2, especially in the presence of its substrate Rab29 which stimulates LRRK2 recruitment and its kinase activity [39]. LRRK2 recruitment to the TGN is mediated *via* Rab29, increasing the autophosphorylation of LRRK2 and results in membrane stabilization of activated Rab proteins [19]. Moreover, LRRK2 interacts with vacuolar protein sorting protein 52 (VPS52), a protein implicated in the retrograde transport of proteins from endosomes to the Golgi apparatus. This interaction is important for intracellular transport, especially soma-TGN transport [39]. LRRK2 insufficiency can lead to defects in trans-Golgi to lysosome trafficking and vesicular transport abnormalities which is associated with destruction of endocytic cargo [40]. Casein kinase 1α (CK1α)-mediated phosphorylation of LRRK2 is crucial for the recruitment of LRRK2 to TGN46 carrying vesicles and regulation of TGN clustering [41]. In addition, vesicles derived from Golgi are actively involved in autophagosome production. It has been previously established that mutant LRRK2 is associated with abnormal Golgi-to-autophagosome transport that is restored after inhibiting LRRK2 activity [42,43]. Consequently, the TGN is an essential hub where disturbances caused by LRRK2 abnormalities can simultaneously affect various intracellular trafficking pathways.

## Physiological role of LRRK2 in mitochondria

Dysfunction of mitochondria, which are organelles that play a key role in cellular respiration and the production of energy, has been linked to LRRK2 abnormalities. Several previous studies have uncovered the role of LRRK2 in mitochondrial dynamics, mitophagy and control of mitochondrial survival, with its disruption leading to different pathologies. Genetic variation in LRRK2, such as R1441C is associated with abnormal mitochondrial function, demonstrated by reduced mitochondrial membrane potential, abnormal morphology, and impaired mitophagy. These abnormalities are connected to reduced PINK1-dependent pS65Ub deposition and MIRO1 degradation, which are critical for stability and transport of mitochondria [31,32,44]. Moreover, mitochondrial homeostasis in kidneys is regulated by LRRK2, as its expression is associated with MFN2 destruction, worsening destruction of mitochondria during acute kidney injury. On the other hand, LRRK2 insufficiency leads to reduced mitochondrial damage and oxidative stress in kidney tissues [45]. The mitochondrial respiration activity has been affected by Roco4 and LRRK2 mutations. It has been shown that LRRK2 affects mitochondrial function *via* indirect pathways and this effect depends on other cytosolic cofactors [46]. Pathogenic G2019S variation confers greater susceptibility to mitochondrial toxins [47], leading to damage of dopaminergic neurons of patients carrying LRRK2 mutations [48]. Two proposed (and maybe overlapping) mechanisms by which LRRK2 variants may lead to mitochondrial dysfunction and pathologies, including direct damage to mitochondria, making them more vulnerable to toxins [49] and compromised cell capacity to handle malfunctioning mitochondria [50].

LRRK2 is also employed in ER–mitochondrial tethering, which is essential for proper mitochondrial function. The activity of E3 ubiquitin ligases (MARCH5, MULAN, Parkin) is regulated by kinase activity of LRRK2. Mutant LRRK2 dissociates from these proteins resulting in enhanced ubiquitin-dependent destruction of ER–mitochondrial tethering proteins, resulting in impaired mitochondrial bioenergetics. By contrast, kinase-dead LRRK2 binds E3 ligases, preventing their activation and protecting tethering proteins from degradation [51]. Mitochondrial DNA damage becomes more prevalent in LRRK2 mutations, which can be reversed by genetic repair of these mutations or inhibition of LRRK2 kinase activity [52–54]. LRRK2 regulates mitochondrial calcium homeostasis through its functional interaction with the mitochondrial Na^+^/Ca^2+^/Li^+^ exchanger (NCLX). NCLX is the primary mediator of Ca^2+^ efflux in the mitochondria. Hyperactivation of LRRK2 kinase activity results in impaired NCLX-dependent calcium extrusion, resulting in retention of mitochondrial Ca^2+^, increased ROS production, sensitization of the mitochondrial permeability transition pore (mPTP), membrane depolarization, and activation of cell death pathways [55]. Thus, defects in LRRK2 are associated with compromised mitochondrial integrity which is NCLX-mediated calcium efflux-dependent, linking dysfunction of LRRK2 kinase to mitochondrial stress and neurodegenerative vulnerability.

## Physiological role of LRRK2 in endoplasmic reticulum

The endoplasmic reticulum (ER), a crucial organelle for calcium storage, peptide synthesis, folding, and trafficking, has been recognized as an important location for LRRK2 activity. Recent studies have investigated how LRRK2 impacts the structure and function of ER, and its interactions with other organelles, which may affect homeostasis. LRRK2 connects with proteins that shape the ER, such as reticulons, to preserve the ER integrity [56]. Additionally, LRRK2 is essential for interaction of ER with other cellular organelles. For instance, ER-mitochondrial interaction is regulated by LRRK2 *via* controlling the function of tethering proteins including MARCH5, MULAN, and Parkin through kinase-dependent protein-protein interactions. Genetic disruption of LRRK2 is associated with increased degradation of these ligases, resulting in impaired ER-mitochondrial connection and mitochondrial activity [51]. LRRK2 is also found to be involved in ER-Golgi trafficking, where it enhances anterograde vesicles trafficking [57]. Furthermore, LRRK2 is required for ER-lysosome interaction. It mediates lysosomal tubulation and sorting where the interaction with ER is necessary to complete the process. Changes in the morphology of ER *via* a decrease in ER tubules is associated with a decrease in LRRK2-mediated sorting of lysosomes [58]. Previous studies have reported that LRRK2 is involved in ER activity in dopaminergic neurons, including the response to stress and protein folding. It upregulates the expression of a key ER-related chaperone required for stress response, called GRP78 [59]. Astrocytes with PD-linked LRRK2 mutant have shown accelerated ER stress and cell death through ER Ca2^+^ depletion. This observation was also linked to mitochondrial dysfunction caused by Ca2^+^ overload [60]. The role of LRRK2 in ER stress is connected to its multifaceted positions in endocytosis, autophagy and cellular organelles equilibrium [61].

## Physiological role of LRRK2 in translation system control

LRRK2 plays a significant role in the regulation of translation and cellular responses. Recent studies highlight the activity of LRRK2 in controlling protein synthesis and its association with various cellular structures, especially in stressful situations. Disruption of LRRK2 in neurons results in increased overall protein translation, suggesting a negative regulatory role for LRRK2 [62]. In translation systems, LRRK2 has a direct interaction with uMtCK, inhibiting its processing and entry to mitochondria. This connection promotes ANT-VDAC binding, resulting in opening of permeability transition pores, release of Cyto C and induction of apoptosis [63]. Moreover, LRRK2 has a role in translational control, namely boosting the translation of mRNAs that have complex 5′ untranslated region structures. Dysregulated translation is observed in LRRK2 pathogenic variants, which contributes to impaired calcium homeostasis in dopaminergic neurons [64].

LRRK2 regulates protein synthesis *via* its activity in ribosomes. Although minimal or no phosphorylation of human 4E-BP by LRRK2 was observed [65], LRRK2 has mediated 4E-BP phosphorylation in other models, resulting in enhanced cap-dependent translation and dopaminergic neurotoxicity [66]. Additionally, small fractions of ribosomal proteins such as S11, S15, and S27 are phosphorylated by LRRK2. S15 phosphorylation improves global translation while phosphodeficient S15 rescues LRRK2 variant-mediated neurotoxicity [67]. Moreover, evidence suggests that LRRK2 is potentially involved in translation machinery such as regulation by microRNA (miRNA). LRRK2 interacts with Argonaute 1 (Ago1), and LRRK2 mutations results in downregulation of Ago1-a key miRNA effector- inhibiting some miRNAs activity and stimulating their cell cycle-related targets, DP1 and E2F1. These changes are associated with neurotoxicity which are reversed by increased miRNA activity and decreased DP1 and E2F1 [68]. Recently, it has been shown that G2019S LRRK2 disrupts the translation of genes involved in calcium homeostasis, including voltage-gated calcium channels subunits, resulting in high calcium levels intracellularly. This pathogenic LRRK2- associated abnormal translation copresents with increased translation of mRNAs with complex 5’UTR secondary structure [69].

## Physiological role of LRRK2 in cytoskeleton dynamics

LRRK2 regulates the dynamics of the cytoskeleton *via* its GTPase/kinase activity by interaction with diverse target proteins and phosphorylation of Rab GTPases to conserve structural integrity and function of the cytoskeleton [70]. LRRK2 has an important role in cytoskeletal dynamics, notably in neuronal cells, where it regulates a variety of cellular processes required for neuronal survival and activity. LRRK2 is required for maturation of oocyte, since it affects organization of spindles and alignment of chromosomes, along with oxidative stress through disruption of mitochondria distribution and function. Inhibiting LRRK2 activity reduces actin levels in both cytoplasmic and cortical areas, demonstrating its function in cytoskeletal dynamics during oocyte maturation [71]. LRRK2 interaction with constituents of the cytoskeleton in neurons is essential for vesicular transport and synaptic plasticity. Improved LRRK2 connection with actin cytoskeleton-associated proteins is mediated *via* Ser395 phosphorylation. This interaction is important for development of BDNF-mediated synaptic processes [72,73]. LRRKE2 also interacts with centrosomes and microtubules required for activities such as neurite development, intracellular transport, and ciliogenesis, emphasizing its vital role in cytoskeleton organization and compartmentalization [74].

LRRK2 binds directly to β-tubulin isoforms (TUBB, TUBB4, and TUBB6). This connection potentially affects microtubule stability [75,76]. Its connection with actin has also been identified. LRRK2 phosphorylates Ezrin, Radixin, and Moesin (ERM), which connect the actin cytoskeleton to the plasma membrane. The G2019S mutation stimulates phosphorylation of ERM proteins, increasing filopodial F-actin accumulation and limiting neurite elongation. On the other hand, LRRK2 knockout lowers phosphorylated ERM and F-actin levels, improving neuronal cell elongation. These findings support the role of LRRK2-ERM signalling dynamics of the cytoskeleton during cellular development [77]. Pathogenic LRRK2 mutations disrupt axonal transport and motor function by preferentially binding to deacetylated microtubules while restoring microtubule acetylation rescues axonal transport impairment and locomotor abnormalities [75].

LRRK2-mediated phosphorylation of Rab GTPases has been linked to alterations in microtubule-dependent trafficking processes. Phosphorylated Rab10 mainly interacts with proteins involved in cilia formation. LRRK2 mutations are associated with faulty cilia formation and Sonic Hedgehog (Shh) signalling, that have been reversed by inhibiting kinase activity of LRRK2 [78]. The function of centrosomes is also affected by LRRK2 activity. It has been reported that pathogenic LRRK2 variants are associated with, centrosomal mispositioning and split centrosomes resulting from pericentrosomal phosphorylated Rab8a accumulation. This phenotype was reversed by blocked LRRK2 kinase activity [79,80].

The physiological role of LRRK2 in organelles and cellular processes is summarized in Table 1.

**Table 1.** The physiological role of LRRK2 in organelles and cellular processes.

Organelle/cellular system	Key Roles of LRRK2	Mechanism / Molecular Partners	Consequences	References
Autophagy/Lysosome	Formation of autophagosome and phagophore, fusion of autophagosome-lysosome, maturation of lysosomes, destruction of lysosomal protein, and regulation of lysosomal pH and calcium homeostasis. Regulation of chaperone-mediated autophagy (CMA) and mitophagy.	Rab GTPases: Rab1, Rab3, Rab5, Rab8, Rab10, Rab12, Rab29, Rab35, and Rab43. Vacuolar-type H+-ATPase pump a1 subunit. EHBP1/EHBP1L1. LAMP2A multimerization.	Impaired clearance of misfolded proteins. Oxidative stress.	[14–24,26–32]
Endocytosis & trans-Golgi Network (TGN)	Controls vesicle trafficking, endosomal maturation, cargo sorting, amphisomes formation and recycling to Golgi.	Rab GTPases: Rab5, Rab7L1, Rab8a, Rab10, and Rab29. Clathrin. Dynamin and endophilin A. Vacuolar protein sorting protein 52 (VPS52). Casein kinase 1α (CK1α).	Golgi disintegration. Faulty autophagy. Impaired synaptic vesicle trafficking and recycling.	[18,19, 33–43]
Mitochondria	Modulates mitochondrial fission/fusion, mitophagy, bioenergetics, Calcium homeostasis and ROS stress response. Regulates ER–mitochondrial tethering.	PINK1. MIRO1. MFN2. E3 ubiquitin ligases: MARCH5, MULAN, and Parkin. Na+/Ca2+/Li + exchanger.	Reduced mitochondrial membrane potential. Abnormal morphology. Impaired mitophagy. Mitochondrial fragmentation. Mitochondrial DNA damage. Oxidative stress.	[31,32, 44–55]
Endoplasmic Reticulum	Maintains integrity of ER morphology. Modulates ER interaction of ER with other organelles (Mitochondria, Golgi trafficking and lysosomes). Involved in ER stress response.	Reticulons. MARCH5. MULAN. Parkin. GRP78.	Disruption of endocytosis, autophagy and cellular organelles equilibrium.	[51,56–61]
Translation system	Controls protein synthesis. Modulates apoptosis. Controls ribosome function and assembly. Regulates translation of structured mRNAs. Regulates translation of genes involved in calcium homeostasis.	uMtCK. ANT-VDAC. Cyto C. Phosphorylation of 4E-BP. Phosphorylation of S11, S15 and S27. Argonaute 1. DP1 and E2F1.	Impaired protein synthesis and calcium homeostasis.	[62–69]
Cytoskeleton dynamics	Controls actin, tubulin and microtubule dynamics. Regulates vesicle motility and axonal transport.	GTPase/kinase activity. Actin-associated proteins Connects to centrosomes and microtubules. TUBB, TUBB4 and TUBB6 Phosphorylation of Ezrin, Radixin, and Moesin. Rab10	Impaired neurite development. Deficient intracellular transport and ciliogenesis.	[70–80]

## LRRK2 gene expression and regulation

LRRK2 is highly expressed in both central and peripheral organs, and its location suggests that it may play an essential role in systemic disorders and NDs. LRRK2 is constitutively expressed throughout the human brain in both neuronal and glial populations, including astrocytes and microglia. Notably, it is particularly enriched in immune-competent glial cells (microglia), where it plays a central role in regulating neuroinflammatory responses, especially under conditions of cellular stress. Although LRRK2 is present in neurons—particularly at synaptic sites—its expression levels are generally higher in glial cells than in neuronal cells [81–83].

In dopaminergic neurons, while the substantia nigra shows considerable expression levels of LRRK2, the majority of dopaminergic neurons in the ventral tegmental region hardly express this protein [84]. Early studies reported that in addition to the midbrain, non-dopaminergic areas like the cerebral cortex, hippocampus, medulla, spinal cord, cerebellum, and putamen likewise express LRRK2. Interestingly, strong expression of LRRK2 has been identified in the striatum, which is the main dopaminergic projection target [7,84–87]. However, recent studies have confirmed the widespread distribution of LRRK2 across multiple brain regions and neural cell populations [88,89], supporting its diverse roles in neuronal signalling and vesicular trafficking within the central nervous system.

**Figure 2.**
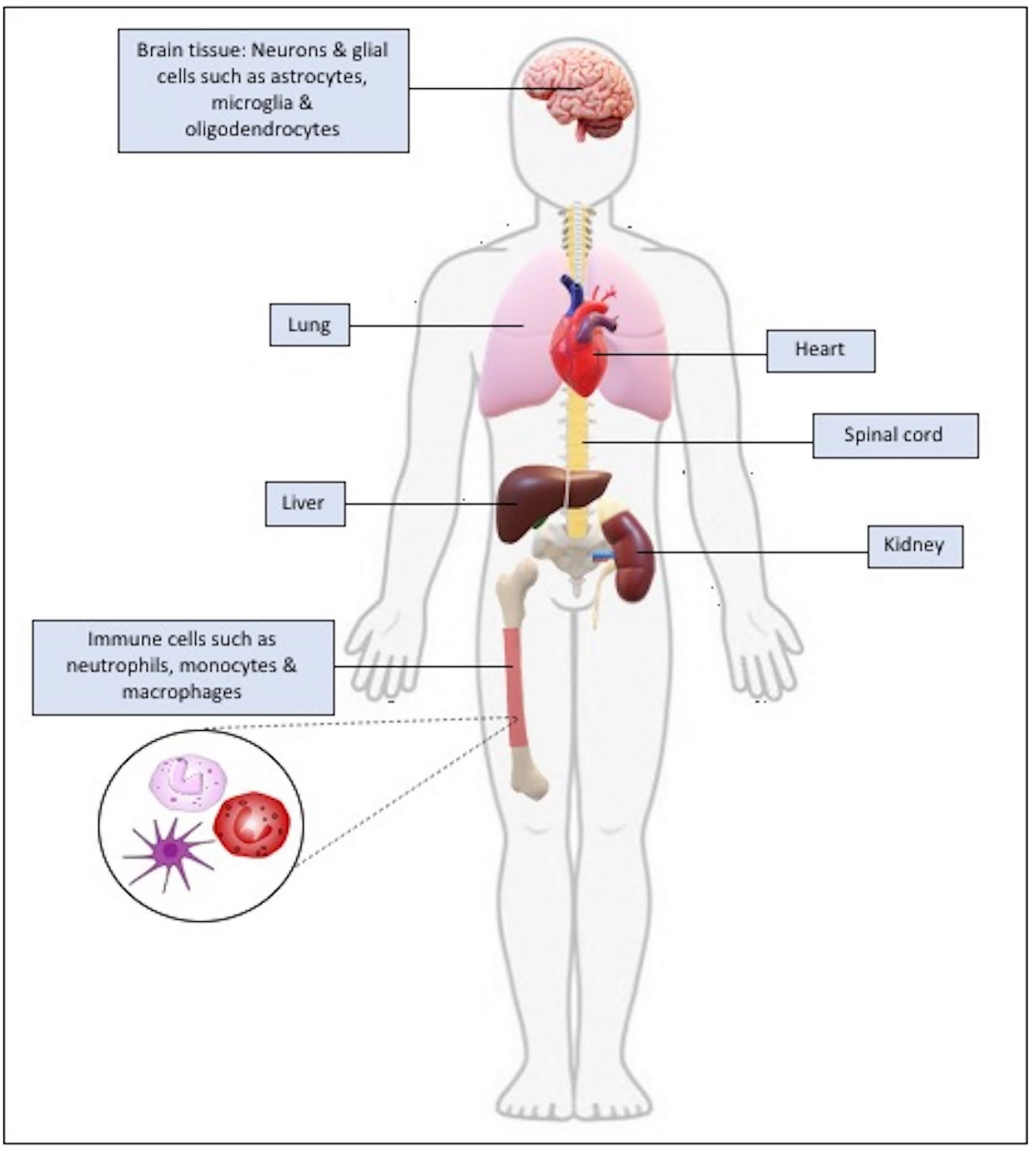
Tissue- and cell-specific expression of Leucine-rich repeat kinase 2 (LRRK2) Across Brain and Peripheral Tissues. LRRK2 is widely expressed across the body in different cell types and tissues, including high levels of expression in brain regions, lung, kidney, and peripheral immune system.

Similarly, LRRK2 is extensively expressed in peripheral organs outside of the central nervous system (CNS), such as the liver, heart, kidneys, and lungs—especially in the renal cortex and alveolar type II epithelial cells that regulate pulmonary homeostasis [90,91]. Its maximum peripheral expression is also shown in immune cells, particularly in CD14^+^CD16^+^ pro-inflammatory monocytes, neutrophils, monocytes, and dendritic cells [92,93]. Immunological stimuli, such as lipopolysaccharide (LPS) [94], interleukin-1β (IL-1β) [95], and interferon-γ (IFN-γ) [96] further increase expression in various cell types, underscoring the importance of LRRK2 in inflammation and immunological responses. The expression patterns of LRRK2 across human body is shown in Figure 2.

The expression of LRRK2 is strictly controlled, at both transcriptional and post-transcriptional levels. Sp1 an ubiquitously expressed zinc-finger transcription factor that binds to GC-rich regions in gene promoters, activating or repressing genes involved in fundamental processes like cell growth, differentiation, immune response, and DNA repair, is a major enhancer of LRRK2’s promoter activity. mRNA levels of LRRK2 are increased with increased expression of the transcription factor Sp1 and decreased with Sp1 pharmacological inhibition [97]. At the post-translational level, LRRK2 is phosphorylated at a number of conserved regions, including Ser910, Ser935, Ser955, and Ser973 in order to stabilise its interaction with 14-3-3 proteins. This connection is broken and LRRK2 ubiquitination and degradation are encouraged when LRRK2 kinase activity is inhibited, resulting in dephosphorylation at Ser935 [98]. Regulation of LRRK2 expression and structure is essential for modulating its function in the body, the different regulatory modalities may explain the different roles of LRRK2 in health and disease, depending on the context. Based on its structure, LRRK2 contains two catalytic domains: a serine/threonine kinase domain and a ROC GTPase domain [9]. The kinase domain mediates autophosphorylation as well as phosphorylation of downstream substrates. The GTPase function is conferred by ROC domain, which together with the COR domain, create the ROC:COR assembly, regulating the LRRK2 activity [9]. Multiple protein-protein interaction domains (ARM, ANK, LRR, and WD40) are also present within the structure of LRRK2, indicating that it is involved in vast signalling networks. LRRK2 may switch between inactive monomeric and active dimeric assemblies. Dimerization of this protein is associated with increased kinase activity on biological membranes [61].

A significant amount of attention has been directed towards kinase activity of this protein because of the G2019S mutation. G2019S increases phosphorylation and has been associated with PD [1]. Rab GTPases (such as Rab3, Rab8, Rab10, Rab12, Rab29, Rab35 and Rab43), that modulate vesicle trafficking and cilia assembly, are phosphorylated by LRRK2 [12]. Some disease-associated LRRK2 variants exhibit enhanced Rab phosphorylation despite showing minimal or no substantial change in intrinsic kinase activity—potentially by enhancing target interaction or membrane connection [99]. Rab29 serves as an important modulatory control by attracting LRRK2 to the Golgi, resulting in increased kinase activity and Rab phosphorylation. Surprisingly, phosphorylation of Rab29 may prevent more LRRK2 activation, implying a feedback loop [19]. These observations support kinase inhibitors as prospective treatment options. On the other hand, the GTPase activity of LRRK2 is understudied but still crucial. Previous research has established that PD-associated mutations located in the ROC:COR domain boost GTP binding or diminish GTP hydrolysis, maintaining LRRK2 activity and GTP-binding [100]. This observation was supported by the finding that PD-protective variants, such as R1398H, perform the opposite [101]. In contrast to other classical GTPases, the traditional GEFs and GAPs are absent in LRRK2. LRRK2 is proposed to operate through a GTPase-activated-by-dimerization (GAD) mechanism, whereby nucleotide binding influences its oligomeric state. In this model, specific nucleotide-bound conformations promote dimer formation, which is associated with enhanced kinase activity, whereas alternative nucleotide states favour monomerization and reduced activity [99]. Thus, changes in LRRK2 assembly are thought to play an important role in regulating its kinase function. Some pathologic variants are associated with GTP-bound dimers, producing sustained kinase activity [102].

LRRK2 activity is dependent on a precisely regulated cycle of kinase activation, GTP hydrolysis, and dimerization. For example, LRRK2 that is in dimeric state and membrane-localized has kinase activity, especially when recruited by GTP-bound Rab29. On the other hand, GTP hydrolysis is associated with conformational transitions toward a less active, monomeric configuration [99]. It can be considered that Rab29 enhances kinase activity *via* binding and stabilizing LRRK2 dimers, whereas GTPase function regulates the extent of activation. Disease causing mutations accelerate this loop yielding hyperphosphorylated Rab and possibly resulting in PD development [61].

## Mutations of LRRK2 in PD

*LRRK2* gene is the most regularly mutated gene in familial PD and is linked to sporadic cases too. *LRRK2* mutations that are linked to PD usually result in changed catalytic function of the enzyme and unusual protein-protein interactions, leading to degeneration of dopaminergic neurons [6]. There are multiple variants of *LRRK2* mutation that are linked to PD, such as G2019S located in the kinase domain which is the most common cause of sporadic and familial PD [6]. The carriers of G2019S mutation show almost the same clinical features as patients with other *LRRK2* mutations, with overall age of onset being around 60 years [103]. This mutation is associated with hyperactivity of kinase activity in the cells [1]. Another major variant is R1441C/G/H that is located in the ROC domain and is associated with reduced GTP hydrolysis and improved kinase function [104]. More mutations in ROC domain (e.g. Y1699C), and WD40 domain (e.g. G2385R) have been reported as risk variants or pathogenic for PD and are also associated with modulation in kinase and GTPase activity of the LRRK2 protein [105,106]. In addition, several other variants across different LRRK2 domains have been recognized in different PD patients. However, the functional implication of those mutations in the pathogenesis of PD are yet to be confirmed. Table 2 summarizes LRRK2 mutations that are linked to PD, sorted by the affected domains and functional impact. The specific role of pathogenic variants of LRRK2 in PD will be discussed in more details in the following sections.

**Table 2.** Examples of LRRK2 variants associated with PD, clinical classification and functional effects.

Mutation	Domain	Clinical classification	Functional impact	References
A211V	ARM	VUS	↑ kinase activity	[182,183]
H230R		VUS	↑ kinase activity	[184]
A397T		NA	↑ kinase activity	[185]
G472R		NA	↑ kinase activity	[185]
K544E		VUS	Unknown	[183]
L550W		NA	↑ kinase activity	[185]
N551K		NA	Unknown	[186]
P755L	ANK	NA	Unknown	[186]
R767H		Likely pathogenic	↑ kinase activity	[187]
Q923H	LRR	VUS	Unknown	[186]
S973N		VUS	Unknown	[186]
R1067Q		Likely pathogenic	↑ kinase activity	[187]
I1371V		NA	Unknown	[186]
T1410M		NA	Unknown	[186]
A1440P	ROC (GTPase)	VUS	↑ kinase activity	[184]
N1437H		Pathogenic	↓ GTPase activity	[186]
R1441C		Pathogenic	↓ GTPase activity → ↑ kinase activity	[186]
R1441G		Pathogenic	↓ GTPase activity	[186]
R1441H		Pathogenic	↓ GTPase activity	[186]
A1442P		Pathogenic	Destabilize the ROC: CORB interface	[187]
V1447M		Pathogenic	Destabilize the ROC: CORB interface	[187]
A1464G		VUS	Unknown	[187]
S1508R		NA	Unknown	[187]
R1514Q	COR	NA	Unknown	[187]
R1628P		NA	Destabilize the CORA	[187]
Y1699C		Pathogenic	Destabilizes ROC–COR interface → ↑ kinase activity	[187]
R1725Q		VUS	Unknown	[186]
R1728H/L		Likely pathogenic	Destabilize COR: COR dimer interface	[187]
S1761R		Likely pathogenic	destabilize the CORB	[187]
M1869T		VUS	Unknown	[186]
D1887G	Kinase	NA	↑ kinase activity	[185]
I1991V		VUS	Unknown	[186]
I2012T		VUS	Unknown	[186]
G2019S		Pathogenic	↑ Kinase activity	[186]
I2020T		Pathogenic	Alters kinase domain conformation	[186]
D2175H	WD40	VUS	Disrupted structural integrity	[188]
T2356I		VUS	Disrupted structural integrity	[188]
L2439I		VUS	Disrupted structural integrity	[188]
G2385R		NA	↑ Kinase activity	[188]

NA: not available, VUS: Variant of unknown significance.

### Mechanisms and pathological role of LRRK2 in PD

**Figure 3.**
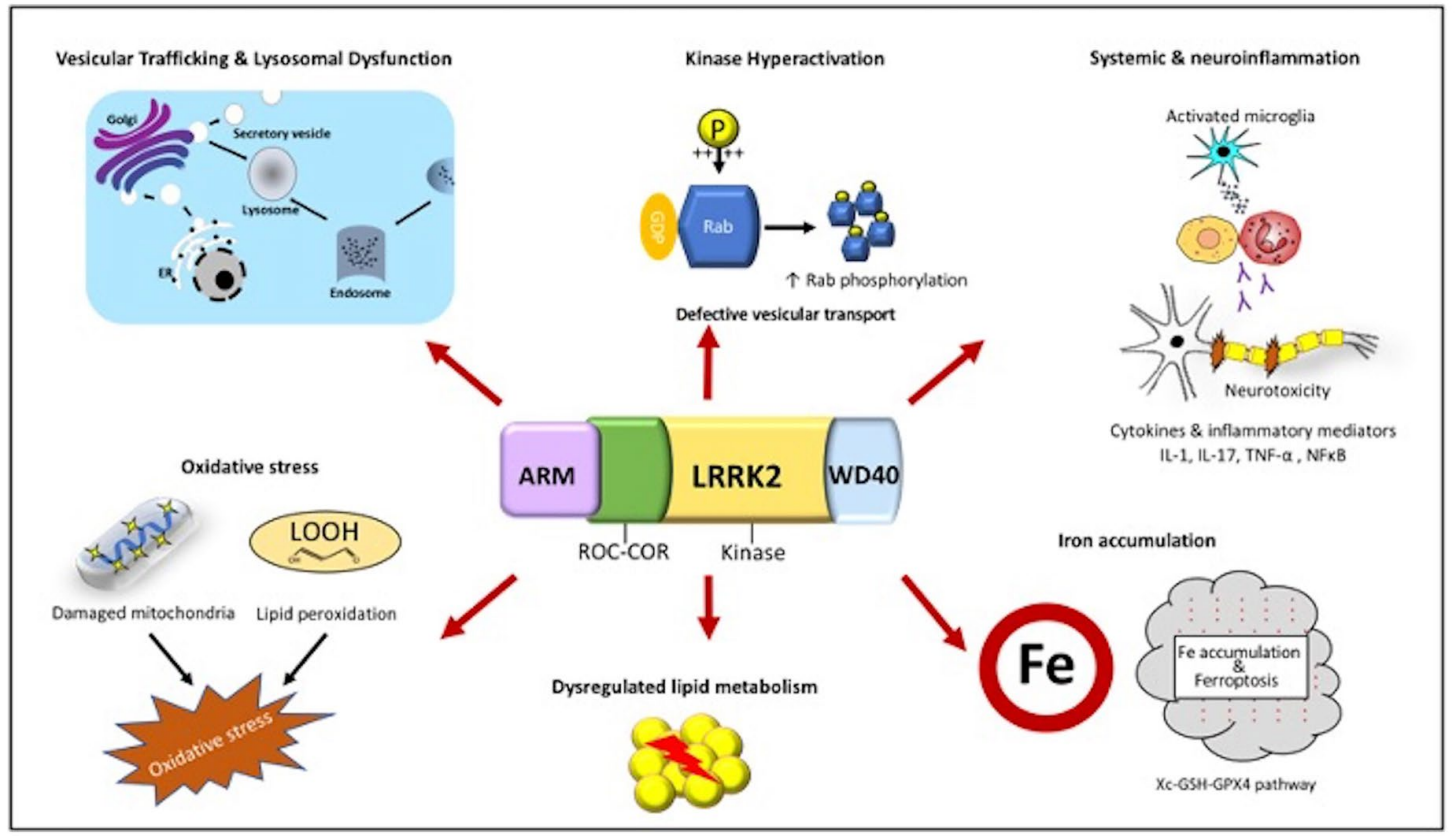
Schematic diagram illustrating pathogenic mechanisms of Leucine-rich repeat kinase 2 (LRRK2) in Parkinson’s disease. The diagram shows a summary of the major cellular and molecular pathways by which LRRK2 contributes to PD pathogenesis. Collectively, these interconnected mechanisms converge on neuronal death, α-synuclein aggregation, and dopaminergic neurons degeneration, forming a pathogenic network linking peripheral inflammation, mitochondrial impairment, and lysosomal dysfunction in LRRK2-associated PD. Abbreviations: ARM; Armadillo domain, ER, endoplasmic reticulum; GDP; guanosine diphosphate, IL, interleukin-1; LRRK2, leucine-rich repeat kinase 2, NF-κB, nuclear factor kappa-B; RCC-COR; Ras of complex, C-terminal of Roc, ROS, reactive oxygen species; TNF-α, tumor necrosis factor alpha.

LRRK2 mutations are responsible for 5–13% of familial PD cases and 1–5% of sporadic PD cases [6]. Several LRRK2 mutations have been identified as genetic risk for PD with G2019S being the most frequent cause of PD, affecting approximately 3–19% of familial PD cases and 1–6% of sporadic PD cases. Beyond genetics, increased activity of LRRK2 is also connected to idiopathic PD, highlighting the broader role of LRRK2 in PD [107]. A characteristic feature of pathogenic LRRK2 in PD is an increased kinase activity, that disrupts cellular structures and pathways required for homeostasis, including lysosomal function, vesicular trafficking, autophagy, and mitochondrial integrity, which will be discussed in the following sections and are summarized in Figure 3.

### Kinase hyperactivation is a key pathogenic feature for PD

Increased kinase function of LRRK2 due to pathogenic variants such as G2019S and R1441C/G/H [1,104] is associated with autophosphorylation and hyperphosphorylation of LRRK2-downstream targets, including Rab GTPases. Increased Rab protein phosphorylation that controls vesicular trafficking results in improper localization and abnormal function of those proteins and impaired cellular function of several organelles. For example, phosphorylation of Rab10 and Rab29 proteins causes lysosomal dysfunction [15]. Radixin and moesin are two proteins that play a key role in regulating neurite growth [108], and are phosphorylation substrates of hyperactive LRRK2. Studies reported that reduced neurite growth is induced by activated G2019S LRRK2 mutant while deletion of LRRK2 and inhibition of phosphorylation has improved neurite growth [77]. Interestingly, the LRRK2 phosphorylation site, Thr558, corresponds to a conserved residue within an identical sequence motif shared by moesin and radixin [109].

Elevated LRRK2 kinase activity is linked to neurodegeneration (ND) of dopaminergic motor neurons. Environmental toxins and mitochondrial stress elevate reactive oxygen species (ROS) levels, which activate LRRK2 kinase activity and enhance phosphorylation of its downstream substrate, Rab10; together with impaired lysosomal function, these events contribute to the accumulation of α-synuclein and the neurodegeneration of dopaminergic motor neurons [110]. On the other hand, pharmacological inhibition of LRRK2 has shown neuroprotective effects against α-synuclein accumulation and ND [111]. Collectively, hyperactivity of kinase function in LRRK2 is a key contributor to ND due to impaired neurite growth, oxidative stress and accumulation of improperly folded proteins.

## Systemic inflammation and neuroinflammation

Neuroinflammation is a characteristic feature in PD, and LRRK2 plays a central role in this process. Since LRRK2 mutations are linked to both familial and sporadic PD, its role in modulating inflammatory responses in the brain and periphery has been investigated in recent studies.

The brain possesses immune privilege due to its strong barrier and multiple protective mechanisms, one of which is inflammation. While inflammation serves a critical defence response that helps clear out pathogens, it also contributes to neurotoxicity and degeneration when excessive, explaining its dual role in the CNS. Studies have revealed that brain inflammation driven by activated microglia is one of the manifestations observed in carriers of the LRRK2 mutation, even among non-symptomatic carriers [112]. Therefore, the preliminary assumption that inflammatory response in the CNS is triggered by ND, instead of being an active participant in the process needs to be re-evaluated [113]. Peripheral cytokines and inflammatory mediators can also trigger inflammatory response in the CNS, leading to ND [114]. Impaired blood brain barrier integrity observed in PD patients is potentially a contributing factor in this situation [115]. Cell infiltration into CNS, such as T lymphocytes has been documented in post-mortem tissues of PD victims. This infiltration aggravates neuroinflammatory response and ND through IL-7 receptor and NFκB pathway activation [116]. The interaction between PNS and CNS suggests that peripheral infection might also predispose to PD pathogenesis [117].

In addition to PD, LRRK2 is also linked to several inflammatory diseases (such as Leprosy and Crohn’s disease) [118]. This connection supports its wider involvement in controlling inflammatory processes. Remarkably, a higher association between having inflammatory bowel disease (IBD) and developing PD was observed [119], with anti-tumor necrosis factor (anti-TNF) therapy for IBD not been shown to increase individual’s risk of acquiring PD. These findings potentially support the assumption that PD might be induced by peripheral inflammation, and interventions targeting inflammatory response might have a protective effect against PD development.

More findings supporting the role of inflammation in developing PD have been documented. For example, higher levels of cytokines, microglia, and astrocyte proliferation have been found in the serum or cerebrospinal fluid (CSF) of PD’s patients. Furthermore, PD’s patients with G2019S mutation displayed higher levels of inflammatory mediators, such as IL1 [120]. Post-mortem brain tissues of PD displayed inflammatory response induced by microglia. Microglial immune response-neurotoxicity is mediated *via* microglial Fcγ receptor in PD. Microglia that has positive Fcγ receptor is stimulated by PD patients’ immunoglobulins while this response is absent with the absence of Fcγ receptor, with no neurotoxicity being observed [121]. Moreover, the G2019S mutation is associated with decreased microglial motility [122], suggesting that microglial behaviour is affected by LRRK2 mutations. Higher expression levels of LRRK2 are detected centrally in microglia and peripherally in monocytes and macrophages, emphasizing further its role in the inflation.

Activation of toll-like receptor 4 (TLR4) enhances expression of LRRK2 and its kinase function [123]. LRRK2 kinase hyperactivity is related to excessive release of pro-inflammatory cytokines, such as IL-1β and TNF-α [120,123]. It has been also found that LRRK2 mutations are associated with neuroinflammation that is mediated *via* NF-κB signalling, modulating inflammatory response and cytokine release. It can activate microglial function *via* regulation of NF-κB signalling pathway and the system Xc-GSH-GPX4 pathway, which are involved in neuroinflammation and ferroptosis [124]. On the other hand, inactivation of LRRK2 is associated with protective effects against neurotoxicity *via* inhibition of neuroinflammation and increased production of neuroprotective factors [124]. Another recent study has reported that LRRK2 controls microglial-mediated neuroinflammation and ferroptosis through the p62-Keap1-Nrf2 pathway. Inhibition of LRRK2 activity relieves neuroinflammation by reducing the release of cytokines, and activation of p62-Keap1-Nrf2 pathway, thereby mitigating ferroptosis and oxidative stress [125].

Not only does LRRK2 interact with microglia to promote neuroinflammation, it also interacts with astrocytes exacerbating their role in neuroinflammation stimulated by oligomeric α-synuclein *via* NF-κB pathway. This inflammatory response is suppressed by LRRK2 inactivation [126]. Furthermore, LRRK2 G2019S mutation decreases astrocytic-mediated clearance of α-synuclein, which causes neuronal damage [127].

These observations suggest that LRRK2 modulates microglial and astrocytic activity and influences several cellular signalling pathways involved in inflammation, immune reaction, and the development of ND in PD.

## Lipid metabolism

It is increasingly apparent that LRRK2 mutations are connected to abnormalities in lipid metabolism. LRRK2 plays a key role in vesicle trafficking and lysosomal function that are regulated by lipid dynamics. LRRK2 interacts with the Rab GTPases that control lipid metabolism and trafficking. Carriers of LRRK2 mutations have shown modulation of different types of lipids at different tissues, including sterols, phospholipids, sphingolipids, glycerolipids and fatty acyls, proposing extensive imbalances in lipid metabolism [128]. Recent studies have established how LRRK2 impacts lipid homeostasis and contributes to PD. It has been identified that LRRK2 kinase activity modulates the levels of endolysosomal lipids essential for glycosphingolipid (GSL) breakdown called bis(monoacylglycerol)phosphate (BMP). Accumulation of BMP and GSLs in the CSF and urine of PD patients with LRRK2 mutations has been documented. This is potentially attributed to impaired activity of a PD-linked lysosomal enzyme called glucocerebrosidase (GCase) [129], highlighting the interplay between LRRK2, GCase, and lipid homeostasis [129]. Moreover, Lrrk2 knock-out in mice is associated with significant increase of ceramide levels in brain and modulated sphingolipid composition, suggesting broader disruption of lipid metabolism. It has been also reported that LRRK2 directly interacts with GBA1, the gene that encodes GCase that breaks down glucosylceramide into ceramide [130]. These observations emphasize the idea that LRRK2-PD plays a central role in lipid metabolism and further investigation of LRRK2’s role in lipid homeostasis may offer novel biomarkers and therapeutic targets for PD intervention.

## Vesicular trafficking and lysosomal dysfunction

Mutations of LRRK2 linked to PD are known to significantly impact multiple cellular pathways involved in lysosomal homeostasis, vesicular trafficking and autophagy which are important for maintenance of neuronal integrity and function. Growing bodies of evidence highlight the key role of LRRK2 in controlling different pathways in endolysosmes, such as autophagy, membrane trafficking and protein catabolism. Disturbances in these LRRK2-modulated pathways may contribute to the pathogenesis of PD.

LRRK2 controls membrane trafficking through its connection with Rab GTPases which regulates vesicular trafficking events, including endocytosis, exocytosis, and vesicle recycling. Pathogenic variants of LRRK2 cause phosphorylation of Rab GTPases, such as Rab8A, Rab10, and Rab29, and change their localization into membrane-bound compartments activity and localization [95,131], resulting in impaired synaptic vesicle budding and endocytosis, transport, and fusion events within different cytoplasmic compartments encompassing endosomes, the Golgi apparatus, and synaptic vesicles. Disturbances in vesicular trafficking can lead to synaptic dysregulation *via* impaired release of neurotransmitter and synaptic vesicles recycling that may contribute to ND [132]. In addition, LRRK2 affects axonal trafficking of synaptic vesicles *via* controlling microtubules stability. LRRK2 controls acetylation of tubulin which is important for microtubule stabilization and effective trafficking of vesicles and organelles along the axons [133]. Disruption of such events is associated with deficient cargo delivery peripherally to the synapses and ineffective degradation of cellular components, further leading to cellular stress and neurotoxicity.

Lysosomal homeostasis is critical for clearance of cellular waste products. LRRK2 affects lysosomal function *via* different ways. Mutations of LRRK2 impact lysosomal dynamics and localization. LRRK2 mutations induce Rab7 mediated perinuclear clustering of lysosomes which causes disturbances in autophagosome breakdown [134]. Mutations of LRRK2 cause impaired connection with vacuolar ATPase (v-ATPase) complex components, especially the a1 subunit that maintains the acidic pH of lysosomes. This impaired connection results in abnormal lysosomal enzyme trafficking and acidification, impaired autophagosome-lysosome fusion, and impaired cellular ability to clear cellular cargo [14]. These lysosomal abnormalities are associated with impaired proteostasis and may promote the accumulation of toxic α-synuclein, which is commonly observed in PD pathology, although not all LRRK2-associated cases exhibit Lewy pathology.

The role of LRRK2 in PD is remarkably affected by other PD-related proteins, such as GCase, encoded by GBA1 gene. The two proteins can regulate each other’s function and are involved in lysosomal function and autophagy. PD-LRRK2 mutations are associated with inhibition of Case which leads to impaired lysosomal function and α-synuclein accumulation. Other GBA mutations, disturbing GCase activity also negatively affect autophagy and lysosomal activity regulated by LRRK2, resulting in α-synuclein accumulation. Interestingly, pharmacological inhibition of kinase activity results in enhanced GCase activity, suggesting the pathological role of LRRK2 and GCase in PD [28,135].

The role of LRRK2 in regulating multiple forms of autophagy, including macroautophagy, mitophagy- a selective form of autophagy for degradation of mitochondria, and chaperone-mediated autophagy (CMA), is well established. Pathogenic mutations of LRRK2 disrupt CMA and result in lysosomal precipitation of α-synuclein aggregates impacting cargo translocation [28]. In terms of mitophagy, a study reported that LRRK2 promotes removal of Miro, a regulator of mitochondrial transport, to stimulate mitophagy.

Pathogenic G2019S mutation interferes with this step, impairing mitochondrial degradation and contributing to ND [136]. Likewise, mitophagy is disrupted because of decreased fission of mitochondria and faulty formation of autophagosomes, worsening oxidative stress and neuronal susceptibility [44].

Another study has shown that LRRK2 mutations such as G2019S interfere with CMA of α-synuclein *via* clogging lysosomal binding sites, impairing its degradation. Consequently, cells upregulate CMA receptors as a compensatory response, suggesting a self-sustaining CMA dysfunction that may enhance proteotoxicity induced by α-synuclein [137].

To conclude, disrupted LRRK2 affects multiple intersecting cellular pathways that are required for neuronal integrity, including vesicle trafficking, lysosomal homeostasis, and autophagy regulation. These deviations converge on impaired proteostasis and biological molecules transport which may contribute to build up of toxic proteins, mitochondrial death and ultimately, neuronal loss. Deeper insights into LRRK2 interplay with various physiological pathways is critical to develop therapeutic strategies for PD, especially because GCase activators and kinase inhibitors are under clinical evaluation for idiopathic and familial cases of PD.

## Oxidative stress

Impaired mitochondrial homeostasis and increased production of ROS are detected commonly in PD. The LRRK2 implication in mitochondrial activity and oxidative stress and their involvement in the development of PD has been characterized in recent studies. LRRK2 mutations, particularly G2019S, impact the way that cells respond to oxidative stress, usually negatively affecting mitochondrial dynamics and worsening neuronal damage.

Disturbances in mitochondrial homeostasis with increased production of ROS is a characteristic feature in PD. LRRK2 function in mitochondrial dynamics and redox status and their contribution to PD has been established. Mutations of LRRK2, such as G2019S affect the cellular response to oxidative stress with mitochondrial homeostasis and neuronal integrity being affected. For example, LRRK2-mutations linked to PD can interfere with Miro, a protein on the outer mitochondrial membrane required for the proper movement and positioning of mitochondria for mitophagy. Moreover, increased activity of Drp1, a key protein involved in mitochondrial fission, resulting in excessive mitochondrial fragmentation has been linked to LRRK2 mutations [138]. Elevated levels of ROS and reduced mitophagy because of abnormal LRRK2 activity can stimulate oxidative stress and neurotoxicity that can be reversed by antioxidant proteins such as DJ-1 or ERK inhibitors [139–141]. Studies have shown that wild-type LRRK2 stimulates the ERK pathway to alleviate oxidative-stress mediated damage. This protective effect is lost in PD-linked mutations [142]. Furthermore, mutant LRRK2-induced susceptibility to oxidative stress is mediated through inhibition of DAF-16 nuclear translocation, causing downregulation of antioxidant genes, such as *sod-3* and *dod-3* [143]. Moreover, higher levels of oxidative stress markers have been documented in the CSF of asymptomatic LRRK2 mutations carriers [144]. It has been found that pharmacological and genetic suppression of LRRK2 kinase activity reduces ROS build-up and induction of apoptosis, suggesting that LRRK2 has pro-apoptotic function in response to oxidative stress [145,146]. Mechanistically, antioxidant defence mechanisms are inhibited by LRRK2 through downregulation of Nrf2 and its target genes, which is reversed by inhibition of glycogen synthase kinase-3β (GSK-3β) [147]. Additionally, LRRK2 can stimulate generation of ROS through direct phosphorylation and activation of NADPH oxidase 2 (NOX2) [148]. Collectively, these results suggest that under normal conditions, LRRK2 potentiates cells ability to defend oxidative stress, whereas mutant or hyper-active LRRK2 enhances ROS accumulation and cell death. This supports the importance of LRRK2 as a potential treatment target for PD.

## Iron homeostasis

LRRK2 is implicated in iron metabolism through controlling iron transport through lysosomes and endosomes and regulating ferrtinophaghy [149]. Excessive iron accumulation in the substantia nigra is a characteristic feature of PD that has been also identified in LRRK2-Linked PD [124]. *via* LRRK2-mediated phosphorylation of Rab GTPases such as Rab8a, transferrin receptor (TfR) is recycled from endosomes to the cell membrane to maintain sufficient transferrin receptors required for internalizing of transferrin-bound iron [150]. This TfR recycling is inhibited by LRRK2 mutations, resulting in increased intracellular iron accumulation [22,151]. Therefore, iron metabolism, including iron uptake and storage in microglial cells is potentially regulated by LRRK2 in response to neuroinflammatory circumstances [152]. Additionally, ferrous ammonium citrate (FAC) can stimulate the phosphorylation of LRRK2 (S935 and S1292), increasing its activity and enhancing dopaminergic motor neurons uptake of ferrous iron [153]. In summary, dysregulated iron metabolism and iron accumulation induced by LRRK2 can contribute to the development of PD.

## Therapeutic implications of LRRK2 targeting in PD

LRRK2 has been investigated for potential treatment in PD, providing a promising path for developing disease-modifying therapeutic interventions. LRRK2 mutations, specifically G2019S- the most common genetic cause of both familial and sporadic PD- lead to hyperactivation of kinase domain, resulting in ND of dopaminergic motor neuron *via* disturbances in vesicular trafficking, autophagy, lysosomal function, and inflammatory response. Therefore, therapeutic interventions modulating LRRK2 function or expression are being intensely investigated both preclinically and clinically.

Pathological significance of LRRK2 appears from its dual enzymatic functions- GTPase and kinase- that control essential physiological process. The kinase function is enhanced by pathogenic LRRK2 mutations, like G2019S, resulting in abnormal lysosomal function and autophagy, impaired mitochondrial homeostasis, and accumulation of toxic α-synuclein [138,154]. These disturbances make LRRK2 an attractive target in PD, and previous studies have shown that LRRK2 inhibition protects dopaminergic neurons of PD models [155]. Therapeutic targeting of LRRK2 is potentially a safe choice since it has been identified that partial inactivation of LRRK2 is not significantly associated with higher risk of PD development [156,157]. Therefore, developing small molecules and antisense oligonucleotides (ASOs) that affect function and expression of LRRK2 has gained attention in the context of PD management.

## Small-Molecule kinase inhibitors

Advanced therapeutic interventions blocking LRRK2 kinase domain, such as selective small-molecule inhibitors have been explored. DNL201, DNL151 BIIB122, and MLi-2 have shown promising interactions with the target, decreasing Rab phosphorylation and enhanced lysosomal activity in central and peripheral tissues. Early-phase clinical trials have shown that these molecules are generally tolerable, and some of them are proceeding into Phase II and III clinical trials for PD’s drug development [158–160].

Development of reliable biomarkers is essential for monitoring drug response and validating therapeutic strategies. Bis(monoacylglycerol)phosphate (BMP), particularly di-22:6-BMP, has emerged as a robust biomarker of LRRK2 kinase activity in PD. Elevated urinary BMP levels are strongly associated with pathogenic LRRK2 mutations (e.g. G2019S and R1441G/C) that enhance kinase activity, whereas pharmacological inhibition of LRRK2 reduces these levels in both preclinical models and humans [161–165]. In addition, urinary exosome Ser(P)-1292 LRRK2 levels can be detected and correlated to the severity of PD’s clinical presentation [166]. Implicating such biomarkers can help in the evaluation of LRRK2 targeted therapies efficacy.

On the other hand, due to its multifaceted roles intracellularly, systemic blocking of LRRK2 might be associated with harmful consequences such as peripheral organ toxicity (lungs and kidney) and cardiovascular consequences [167], which demands careful observation and dosing during clinical trials. However, some recently engineered compounds have been produced that avoid undesired side-effects [168]. A physiological form of vitamin B12, 5′-deoxyadenosylcobalamin (AdoCbl), has been identified as a novel allosteric inhibitor of LRRK2. Unlike most LRRK2 inhibitors that target the ATP-binding site [169], AdoCbl acts through a non–ATP-competitive mechanism by binding directly to LRRK2, inducing conformational changes that disrupt its dimerization and consequently reduce its kinase activity [170].

## Gene-Silencing strategies

Gene-silencing methods, including RNA interference (RNAi, [171,172]), antisense oligonucleotides (ASOs, [173]), and CRISPR-based approaches [174] can be employed as complementary strategy to directly decrease the synthesis of mutant LRRK2 protein, reducing its neurotoxic effect, especially in PD cases associated with gain-of-function pathogenic mutations like G2019S. ASOs that are restricted to the CNS can help in avoiding peripheral off-target toxicity and have exhibited decreased α-synuclein pathology and reversed abnormal behaviours linked to PD in rodents [173]. Their selective capacity offers safer options than systemic inhibitors to target LRRK2 levels. A bacterial artificial chromosome (BAC)-based homologous recombination system has been employed to generate gene-corrected induced pluripotent stem cells (iPSCs) from patients with PD, carrying G2019S mutation. iPSCs showed typical pluripotency with no off-target effects [175], highlighting the role of genetic correction as a therapeutic option.

## GTPase inhibitors and emerging modalities

Besides the LRRK2 kinase domain, the GTPase enzymatic activity of LRRK2 offers another potential drug target, especially for certain mutation contexts such as R1441C/G. Compounds targeting GTPase domain have the potential to be safer and more selective due to the presence of only four ROCO GTPases in humans, including LRRK1, LRRK2, malignant fibrous histiocytoma-amplified sequence with leucine-rich tandem repeats 1 (MASL1) and death-associated protein kinase (DAPK) 1 [176]. Additionally, this strategy is promising since PD-linked pathogenic mutations increase the prevalence of GTP-bound LRRK2, stimulating its kinase activity and inducing neurotoxicity [177], and targeting the GTP-bound LRRK2 will result in reduced kinase activity and neurotoxicity. Limited number of GTPase inhibitors have been described and are still in the preclinical stages; however, they have shown neuroprotective properties through regulation of LRRK2 dimerization and membrane trafficking networks [178].

## Emerging modalities beyond small molecules

Other than direct inhibitors for LRRK2, novel therapeutic modalities are evolving. Nanobodies blocking LRRK2 kinase function without binding to ATP domain and causing microtubule association have been identified [179]. LRRK2 interactors, such as RGS2 and ArfGAP1 that regulate LRRK2 catalytic function and toxicity can be targeted for treatment of PD. RGS2 controls LRRK2 kinase function and shows neuronal protective properties [180], whereas ArfGAP1 increases GTPase activity of LRRK2 and stimulates LRRK2-mediated reduced neurite length [181].

To conclude, several therapeutic modalities targeting LRRK2 for treatment of PD have been identified (summarized in Table 3) and are promising. Although initial outcomes are inspiring, further studies are required to ensure efficacy and safety of the implicated therapeutic interventions.

**Table 3.** Summary of preclinical and clinical LRRK2-targeted therapeutic strategies in parkinson’s disease.

Therapeutic modality	Example/Tool	Mechanism of action	Therapeutic benefits	References
Small-Molecule Kinase Inhibitors	MLi-2, DNL201, PF-360	Inhibit LRRK2 kinase activity to reduce pathogenic phosphorylation	Reduce α-synuclein aggregation, protect neurons	[158–160]
	ATP-competitive inhibitors	Block ATP-binding pocket to suppress kinase function	Potent inhibition of LRRK2 activity, clinical candidates available	[169]
	RNA interference (RNAi)	Reduce LRRK2 mRNA and protein levels	Restores autophagy, reduces LRRK2 burden	[171,172]
Gene-Silencing Strategies	Antisense oligonucleotides (ASOs)	Bind to mRNA to block translation or promote degradation	Clinically tested, effective reduction of LRRK2	[173]
	CRISPR/Cas9 gene editing	Gene correction or knockout *via* DNA editing	Precise correction of pathogenic mutations	[174]
	Compound 68	Reduce both GTPase and kinase activity of mutant LRRK2	Neuroprotection in G2019S models of PD	[189]
GTPase Inhibitors	Nanobodies	Allosteric inhibition through selective binding to non-ATP sites	Avoid toxicity linked to ATP inhibitors	[179]
	ArfGAP1, RGS2 targeting	Modulate GTPase activity and LRRK2 toxicity through interacting proteins	Enhance or suppress GTPase-dependent toxicity pathways	[190]

## Conclusions and future perspectives

Over the past decade, LRRK2 has emerged as a key regulator of PD pathogenies. Through its kinase and GTPase activities, this protein orchestrates various physiological pathways involved in vesicular trafficking, mitochondrial dynamics, cytoskeletal dynamics, and immune responses. Its dysfunction is associated with ND of brain dopaminergic motor neurons. Despite progress that has been made, there are still gaps present. The mechanisms by which LRRK2 interacts with α-synuclein pathology remains under-investigated. Similarly, how LRRK2 connects peripheral inflammation with central neuroinflammation is still unresolved. The emerging role of LRRK2 in lipid and iron metabolism also broadens the potential area of metabolic biomarkers and intervention targets. Further research is required to outline LRRK2’s-connected networks, characterize its metabolic connections, and introduce therapeutic interventions that focuse on its enzymatic activity and inflammatory mediators. This could help in restoring neuronal homeostasis and propose new therapeutics to cure or delay neurotoxicity associated with PD.

## Data Availability

There is no data associated with this research.

## References

[1] Bouhouche A, Tibar H, Ben El Haj R, et al. LRRK2 G2019S mutation: prevalence and clinical features in moroccans with Parkinson’s Disease. Parkinsons Dis. 2017;2017:2412486–2412487. doi: 10.1155/2017/2412486. 28465860 PMC5390546

[2] Dorsey ER, Elbaz A, Nichols E, et al. Global, regional, and national burden of Parkinson’s disease, 1990–2016: a systematic analysis for the Global Burden of Disease Study 2016. Lancet Neurol. 2018;17(11):939–953. doi: 10.1016/S1474-4422(18)30295-3. 30287051 PMC6191528

[3] Su D, Cui Y, He C, et al. Projections for prevalence of Parkinson’s disease and its driving factors in 195 countries and territories to 2050: modelling study of Global Burden of Disease Study 2021. BMJ. 2025;388:e080952–e080952. doi: 10.1136/bmj-2024-080952. 40044233 PMC11881235

[4] Clarke CE. Parkinson’s disease. BMJ. 2007;335(7617):441–445. doi: 10.1136/bmj.39289.437454.AD. 17762036 PMC1962892

[5] Tanner CM, Ostrem JL. Parkinson’s disease. N Engl J Med. 2024;391(5):442–452. doi: 10.1056/NEJMra2401857. 39083773

[6] Rui Q, Ni H, Li D, et al. The role of LRRK2 in neurodegeneration of Parkinson disease. Curr Neuropharmacol. 2018;16(9):1348–1357. doi: 10.2174/1570159X16666180222165418. 29473513 PMC6251048

[7] Paisán-Ruíz C, Jain S, Evans E, et al. Cloning of the gene containing mutations that cause PARK8-linked Parkinson’s disease. Neuron. 2004;44(4):595–600. doi: 10.1016/j.neuron.2004.10.023. 15541308

[8] Deniston CK, Salogiannis J, Mathea S, et al. Structure of LRRK2 in Parkinson’s disease and model for microtubule interaction. Nature. 2020;588(7837):344–349. doi: 10.1038/s41586-020-2673-2. 32814344 PMC7726071

[9] Deng J, Lewis PA, Greggio E, et al. Structure of the ROC domain from the Parkinson’s disease-associated leucine-rich repeat kinase 2 reveals a dimeric GTPase. Proc Natl Acad Sci USA. 2008;105(5):1499–1504. doi: 10.1073/pnas.0709098105. 18230735 PMC2234173

[10] Marku A, Carrion MDP, Pischedda F, et al. The LRRK2 N-terminal domain influences vesicle trafficking: impact of the E193K variant. Sci Rep. 2020;10(1):3799–3799. doi: 10.1038/s41598-020-60834-5. 32123243 PMC7052203

[11] Biosa A, Trancikova A, Civiero L, et al. GTPase activity regulates kinase activity and cellular phenotypes of Parkinson’s disease-associated LRRK2. Hum Mol Genet. 2013;22(6):1140–1156. doi: 10.1093/hmg/dds522. 23241358

[12] Steger M, Tonelli F, Ito G, et al. Phosphoproteomics reveals that Parkinson’s disease kinase LRRK2 regulates a subset of Rab GTPases. Elife. 2016;5:e12813. doi: 10.7554/eLife.12813. PMC4769169 26824392

[13] Jain BP, Pandey S. WD40 repeat proteins: signalling scaffold with diverse functions. Protein J. 2018;37(5):391–406. doi: 10.1007/s10930-018-9785-7. 30069656

[14] Wallings R, Connor-Robson N, Wade-Martins R. LRRK2 interacts with the vacuolar-type H+-ATPase pump a1 subunit to regulate lysosomal function. Hum Mol Genet. 2019;28(16):2696–2710. doi: 10.1093/hmg/ddz088. 31039583 PMC6687951

[15] Komori T, Kuwahara T. An update on the interplay between LRRK2, Rab GTPases and Parkinson’s disease. Biomolecules. 2023;13(11):1645–1645. doi: 10.3390/biom13111645. 38002327 PMC10669493

[16] Steger M, Diez F, Dhekne HS, et al. Systematic proteomic analysis of LRRK2-mediated Rab GTPase phosphorylation establishes a connection to ciliogenesis. Elife. 2017;6:e31012. doi: 10.7554/eLife.31012. PMC5695910 29125462

[17] Nirujogi RS, Tonelli F, Taylor M, et al. Development of a multiplexed targeted mass spectrometry assay for LRRK2-phosphorylated Rabs and Ser910/Ser935 biomarker sites. Biochem J. 2021;478(2):299–326. doi: 10.1042/BCJ20200930. 33367571 PMC7833208

[18] Purlyte E, Dhekne HS, Sarhan AR, et al. Rab29 activation of the Parkinson’s disease‐associated LRRK2 kinase. Embo J. 2018;37(1):1–18. doi: 10.15252/embj.201798099. 29212815 PMC5753036

[19] Liu Z, Bryant N, Kumaran R, et al. LRRK2 phosphorylates membrane-bound Rabs and is activated by GTP-bound Rab7L1 to promote recruitment to the trans-Golgi network. Hum Mol Genet. 2018;27(2):385–395. doi: 10.1093/hmg/ddx410. 29177506 PMC5886198

[20] Bellucci A, Longhena F, Spillantini MG. The role of Rab proteins in Parkinson’s disease synaptopathy. Biomedicines. 2022;10(8):1941–1941. doi: 10.3390/biomedicines10081941. 36009486 PMC9406004

[21] Eguchi T, Kuwahara T, Sakurai M, et al. LRRK2 and its substrate Rab GTPases are sequentially targeted onto stressed lysosomes and maintain their homeostasis. Proc Natl Acad Sci USA. 2018;115(39):E9115-E9124. doi: 10.1073/pnas.1812196115. PMC6166828 30209220

[22] Mamais A, Kluss JH, Bonet-Ponce L, et al. Mutations in LRRK2 linked to Parkinson disease sequester Rab8a to damaged lysosomes and regulate transferrin-mediated iron uptake in microglia. PLoS Biol. 2022;20(5):e3001621. doi: 10.1371/journal.pbio.3001621. 35507910 PMC9068230

[23] Madureira M, Connor-Robson N, Wade-Martins R. LRRK2: autophagy and lysosomal activity. Front Neurosci. 2020;14:498. doi: 10.3389/fnins.2020.00498. 32523507 PMC7262160

[24] Sun S, Hodel M, Wang X, et al. Macrophage LRRK2 hyperactivity impairs autophagy and induces paneth cell dysfunction. Sci Immunol. 2024;9(101):eadi7907. doi: 10.1126/sciimmunol.adi7907. 39514635 PMC11730131

[25] Eguchi T, Sakurai M, Wang Y, et al. The V-ATPase-ATG16L1 axis recruits LRRK2 to facilitate the lysosomal stress response. J Cell Biol. 2024;223(3):e202302067. doi: 10.1083/jcb.202302067. PMC10791558 38227290

[26] Bentley-DeSousa A, Roczniak-Ferguson A, Ferguson SM. A STING-CASM-GABARAP pathway activates LRRK2 at lysosomes. J Cell Biol. 2025;224(2):e202310150. doi: 10.1083/jcb.202310150. PMC11734622 39812709

[27] Huang T, Sun C, Du F, et al. STING-induced noncanonical autophagy regulates endolysosomal homeostasis. Proc Natl Acad Sci U S A. 2025;122(8):e2415422122. doi: 10.1073/pnas.2415422122. 39982740 PMC11874320

[28] Pang SY-Y, Lo RCN, Ho PW-L, et al. LRRK2, GBA and their interaction in the regulation of autophagy: implications on therapeutics in Parkinson’s disease. Transl Neurodegener. 2022;11(1):5–5. doi: 10.1186/s40035-022-00281-6. 35101134 PMC8805403

[29] Ogata J, Hirao K, Nishioka K, et al. A novel LRRK2 variant p.G2294R in the WD40 domain identified in familial Parkinson’s disease affects LRRK2 protein levels. Int J Mol Sci. 2021;22(7):3708–3708. doi: 10.3390/ijms22073708. 33918221 PMC8038167

[30] Singh F, Prescott AR, Rosewell P, et al. Pharmacological rescue of impaired mitophagy in Parkinson’s disease-related LRRK2 G2019S knock-in mice. Elife. 2021;10:e67604. doi: 10.7554/eLife.67604. PMC8331189 34340748

[31] Wauters F, Cornelissen T, Imberechts D, et al. LRRK2 mutations impair depolarization-induced mitophagy through inhibition of mitochondrial accumulation of RAB10. Autophagy. 2020;16(2):203–222. doi: 10.1080/15548627.2019.1603548. 30945962 PMC6984591

[32] Bonello F, Hassoun S-M, Mouton-Liger F, et al. LRRK2 impairs PINK1/Parkin-dependent mitophagy via its kinase activity: pathologic insights into Parkinson’s disease. Hum Mol Genet. 2019;28(10):1645–1660. doi: 10.1093/hmg/ddz004. 30629163

[33] Arranz AM, Delbroek L, Van Kolen K, et al. LRRK2 functions in synaptic vesicle endocytosis through a kinase-dependent mechanism. J Cell Sci. 2015;128(3):541–52. doi: 10.1242/jcs.158196. 25501810

[34] Matta S, Van Kolen K, da Cunha R, et al. LRRK2 controls an EndoA phosphorylation cycle in synaptic endocytosis. Neuron. 2012;75(6):1008–1021. doi: 10.1016/j.neuron.2012.08.022. 22998870

[35] Rivero-Ríos P, Romo-Lozano M, Fernández B, et al. Distinct roles for rab10 and rab29 in pathogenic LRRK2-mediated endolysosomal trafficking alterations. Cells. 2020;9(7):1719–1719. doi: 10.3390/cells9071719. 32709066 PMC7407826

[36] Heaton GR, Landeck N, Mamais A, et al. Sequential screening nominates the Parkinson’s disease associated kinase LRRK2 as a regulator of Clathrin-mediated endocytosis. Neurobiol Dis. 2020;141:104948–104948. doi: 10.1016/j.nbd.2020.104948. 32434048 PMC7339134

[37] Ravinther AI, Dewadas HD, Tong SR, et al. Molecular pathways involved in LRRK2-linked Parkinson’s disease: a systematic review. Int J Mol Sci. 2022;23(19):11744–11744. doi: 10.3390/ijms231911744. 36233046 PMC9569706

[38] Kuwahara T, Iwatsubo T. The emerging functions of lrrk2 and rab gtpases in the endolysosomal system. Front Neurosci. 2020;14:227. doi: 10.3389/fnins.2020.00227. 32256311 PMC7095371

[39] Wei Y, Awan MUN, Bai L, et al. The function of Golgi apparatus in LRRK2-associated Parkinson’s disease. Front Mol Neurosci. 2023;16:1097633. doi: 10.3389/fnmol.2023.1097633. 36896008 PMC9989030

[40] Lanning NJ, VanOpstall C, Goodall ML, et al. LRRK2 deficiency impairs trans -Golgi to lysosome trafficking and endocytic cargo degradation in human renal proximal tubule epithelial cells. Am J Physiol Renal Physiol. 2018;315(5):F1465–F1477. doi: 10.1152/ajprenal.00009.2018. 30089035 PMC6293309

[41] Chia R, Haddock S, Beilina A, et al. Phosphorylation of LRRK2 by casein kinase 1α regulates trans-Golgi clustering via differential interaction with ARHGEF7. Nat Commun. 2014;5(1):5827–5827. doi: 10.1038/ncomms6827. 25500533 PMC4268884

[42] Vilariño-Güell C, Wider C, Ross OA, et al. VPS35 mutations in Parkinson disease. Am J Hum Genet. 2011;89(1):162–167. doi: 10.1016/j.ajhg.2011.06.001. 21763482 PMC3135796

[43] Beilina A, Rudenko IN, Kaganovich A, et al. Unbiased screen for interactors of leucine-rich repeat kinase 2 supports a common pathway for sporadic and familial Parkinson disease. Proc Natl Acad Sci USA. 2014;111(7):2626–2631. doi: 10.1073/pnas.1318306111. 24510904 PMC3932908

[44] Williamson MG, Madureira M, McGuinness W, et al. Mitochondrial dysfunction and mitophagy defects in LRRK2-R1441C Parkinson’s disease models. Hum Mol Genet. 2023;32(18):2808–2821. doi: 10.1093/hmg/ddad102. 37384414 PMC10481106

[45] Zhang S, Qian S, Liu H, et al. LRRK2 aggravates kidney injury through promoting MFN2 degradation and abnormal mitochondrial integrity. Redox Biol. 2023;66:102860–102860. doi: 10.1016/j.redox.2023.102860. 37633049 PMC10470420

[46] Rosenbusch KE, Oun A, Sanislav O, et al. A conserved role for LRRK2 and Roco proteins in the regulation of mitochondrial activity. Front Cell Dev Biol. 2021;9:734554. doi: 10.3389/fcell.2021.734554. 34568343 PMC8455996

[47] Karuppagounder SS, Xiong Y, Lee Y, et al. LRRK2 G2019S transgenic mice display increased susceptibility to 1-methyl-4-phenyl-1,2,3,6-tetrahydropyridine (MPTP)-mediated neurotoxicity. J Chem Neuroanat. 2016;76(Pt B):90–97. doi: 10.1016/j.jchemneu.2016.01.007. 26808467 PMC4958044

[48] Cooper O, Seo H, Andrabi S, et al. Pharmacological rescue of mitochondrial deficits in iPSC-derived neural cells from patients with familial Parkinson’s disease. Sci Transl Med. 2012;4(141):141ra90. doi: 10.1126/scitranslmed.3003985. PMC3462009 22764206

[49] Angeles DC, Gan B-H, Onstead L, et al. Mutations in LRRK2 increase phosphorylation of peroxiredoxin 3 exacerbating oxidative stress-induced neuronal death. Hum Mutat. 2011;32(12):1390–1397. doi: 10.1002/humu.21582. 21850687

[50] Angeles DC, Ho P, Chua LL, et al. Thiol peroxidases ameliorate LRRK2 mutant-induced mitochondrial and dopaminergic neuronal degeneration in Drosophila. Hum Mol Genet. 2014;23(12):3157–3165. doi: 10.1093/hmg/ddu026. 24459295 PMC4030771

[51] Toyofuku T, Okamoto Y, Ishikawa T, et al. LRRK2 regulates endoplasmic reticulum–mitochondrial tethering through the PERK‐mediated ubiquitination pathway. Embo J. 2020;39(18):e105826. doi: 10.15252/embj.2018100875. 33433003 PMC7507308

[52] Sanders LH, Laganière J, Cooper O, et al. LRRK2 mutations cause mitochondrial DNA damage in iPSC-derived neural cells from Parkinson’s disease patients: reversal by gene correction. Neurobiol Dis. 2014;62:381–386. doi: 10.1016/j.nbd.2013.10.013. 24148854 PMC3877733

[53] Howlett EH, Jensen N, Belmonte F, et al. LRRK2 G2019S-induced mitochondrial DNA damage is LRRK2 kinase dependent and inhibition restores mtDNA integrity in Parkinson’s disease. Hum Mol Genet. 2017;26(22):4340–4351. doi: 10.1093/hmg/ddx320. 28973664 PMC5886254

[54] Gonzalez-Hunt CP, Thacker EA, Toste CM, et al. Mitochondrial DNA damage as a potential biomarker of LRRK2 kinase activity in LRRK2 Parkinson’s disease. Sci Rep. 2020;10(1):17293–17293. doi: 10.1038/s41598-020-74195-6. 33057100 PMC7557909

[55] Ludtmann MHR, Kostic M, Horne A, et al. LRRK2 deficiency induced mitochondrial Ca2+ efflux inhibition can be rescued by Na+/Ca2+/Li + exchanger upregulation. Cell Death Dis. 2019;10(4):265–265. doi: 10.1038/s41419-019-1469-5. 30890692 PMC6424963

[56] Nixon-Abell JJ. Endoplasmic Reticulum Structure and Function in LRRK2-Mediated Parkinson’s Disease; 2017. London.

[57] Cho HJ, Yu J, Xie C, et al. Leucine‐rich repeat kinase 2 regulates Sec16A at ER exit sites to allow ER –Golgi export. Embo J. 2014;33(20):2314–2331. doi: 10.15252/embj.201487807. 25201882 PMC4253522

[58] Bonet-Ponce L, Cookson MR. The endoplasmic reticulum contributes to lysosomal tubulation/sorting driven by LRRK2. Mol Biol Cell. 2022;33(13):ar124. doi: 10.1091/mbc.E22-04-0139. 36044336 PMC9634967

[59] Yuan Y, Cao P, Smith MA, et al. Dysregulated LRRK2 signaling in response to endoplasmic reticulum stress leads to dopaminergic neuron degeneration in C. elegans. PLoS One. 2011;6(8):e22354-e22354. doi: 10.1371/journal.pone.0022354. 21857923 PMC3153934

[60] Lee JH, Han J-H, Kim H, et al. Parkinson’s disease-associated LRRK2-G2019S mutant acts through regulation of SERCA activity to control ER stress in astrocytes. Acta Neuropathol Commun. 2019;7(1):68–68. doi: 10.1186/s40478-019-0716-4. 31046837 PMC6498585

[61] Berwick DC, Heaton GR, Azeggagh S, et al. LRRK2 biology from structure to dysfunction: research progresses, but the themes remain the same. Mol Neurodegener. 2019;14(1):49–49. doi: 10.1186/s13024-019-0344-2. 31864390 PMC6925518

[62] Deshpande P, Flinkman D, Hong Y, et al. Protein synthesis is suppressed in sporadic and familial Parkinson’s disease by LRRK2. Faseb J. 2020;34(11):14217–14233. doi: 10.1096/fj.202001046R. 32926469

[63] Cui J, Yu M, Niu J, et al. Expression of leucine-rich repeat kinase 2 (LRRK2) inhibits the processing of uMtCK to induce cell death in a cell culture model system. Biosci Rep. 2011;31(5):429–437. doi: 10.1042/BSR20100127. 21370995 PMC3971885

[64] Kim JW, Yin X, Martin I, et al. Dysregulated mRNA translation in the G2019S LRRK2 and LRRK2 knock-out mouse brains. eNeuro. 2021;8(6):ENEURO.0310-21.2021. doi: 10.1523/ENEURO.0310-21.2021. PMC8638676 34759048

[65] Trancikova A, Mamais A, Webber PJ, et al. Phosphorylation of 4E-BP1 in the mammalian brain is not altered by LRRK2 expression or pathogenic mutations. PLoS One. 2012;7(10):e47784–e47784. doi: 10.1371/journal.pone.0047784. 23082216 PMC3474772

[66] Imai Y, Gehrke S, Wang H-Q, et al. Phosphorylation of 4E-BP by LRRK2 affects the maintenance of dopaminergic neurons in Drosophila. Embo J. 2008;27(18):2432–2443. doi: 10.1038/emboj.2008.163. 18701920 PMC2543051

[67] Martin I, Kim JW, Lee BD, et al. Ribosomal Protein s15 phosphorylation mediates LRRK2 neurodegeneration in Parkinson’s disease. Cell. 2014;157(2):472–485. doi: 10.1016/j.cell.2014.01.064. 24725412 PMC4040530

[68] Gehrke S, Imai Y, Sokol N, et al. Pathogenic LRRK2 negatively regulates microRNA-mediated translational repression. Nature. 2010;466(7306):637–641. doi: 10.1038/nature09191. 20671708 PMC3049892

[69] Kim JW, Yin X, Jhaldiyal A, et al. Defects in mRNA translation in LRRK2-mutant hiPSC-derived dopaminergic neurons lead to dysregulated calcium homeostasis. Cell Stem Cell. 2020;27(4):633–645.e7. doi: 10.1016/j.stem.2020.08.002. 32846140 PMC7542555

[70] Iannotta L, Greggio E. LRRK2 signaling in neurodegeneration: two decades of progress. Essays Biochem. 2021;65(7):859–872. doi: 10.1042/EBC20210013. 34897411

[71] Wei Y-X, Wang Y-H, Yu X-T, et al. Loss of LRRK2 activity induces cytoskeleton defects and oxidative stress during porcine oocyte maturation. Cell Commun Signal. 2025;23(1):2–2. doi: 10.1186/s12964-024-01997-w. 39748263 PMC11697660

[72] Civiero L, Cogo S, Biosa A, et al. The role of LRRK2 in cytoskeletal dynamics. Biochem Soc Trans. 2018;46(6):1653–1663. doi: 10.1042/BST20180469. 30467120

[73] West AB. LRRK2 regulates synaptic function through BDNF signaling and actin cytoskeleton. 2024.

[74] Eckert M, et al. Proximity proteomics reveals a co-evolved LRRK2-regulatory network linked to centrosomes. 2024. 10.1038/s44319-026-00806-4 PMC13304329 42177293

[75] Godena VK, Brookes-Hocking N, Moller A, et al. Increasing microtubule acetylation rescues axonal transport and locomotor deficits caused by LRRK2 Roc-COR domain mutations. Nat Commun. 2014;5(1):5245–5245. doi: 10.1038/ncomms6245. 25316291 PMC4208097

[76] Law BMH, Spain VA, Leinster VHL, et al. A Direct interaction between leucine-rich repeat kinase 2 and specific β-tubulin isoforms regulates tubulin acetylation. J Biol Chem. 2014;289(2):895–908. doi: 10.1074/jbc.M113.507913. 24275654 PMC3887213

[77] Parisiadou L, Xie C, Cho HJ, et al. Phosphorylation of Ezrin/Radixin/Moesin proteins by LRRK2 promotes the rearrangement of actin cytoskeleton in neuronal morphogenesis. J Neurosci. 2009;29(44):13971–13980. doi: 10.1523/JNEUROSCI.3799-09.2009. 19890007 PMC2807632

[78] Dhekne HS, Yanatori I, Gomez RC, et al. A pathway for Parkinson’s disease LRRK2 kinase to block primary cilia and Sonic hedgehog signaling in the brain. Elife. 2018;7:e40202. doi: 10.7554/eLife.40202. PMC6219843 30398148

[79] Phillips B, Western D, Wang L, et al. Proteome wide association studies of LRRK2 variants identify novel causal and druggable proteins for Parkinson’s disease. NPJ Parkinsons Dis. 2023;9(1):107–107. doi: 10.1038/s41531-023-00555-4. 37422510 PMC10329646

[80] Madero-Pérez J, Fdez E, Fernández B, et al. Parkinson disease-associated mutations in LRRK2 cause centrosomal defects via Rab8a phosphorylation. Mol Neurodegener. 2018;13(1):3–3. doi: 10.1186/s13024-018-0235-y. 29357897 PMC5778812

[81] Iseki T, Imai Y, Hattori N. Is glial dysfunction the key pathogenesis of LRRK2-linked Parkinson’s disease? Biomolecules. 2023;13(1):178. doi: 10.3390/biom13010178. 36671564 PMC9856048

[82] Mutti V, Carini G, Marizzoni M, et al. LRRK2-mediated neuroinflammation-induced neuronal dysfunctions in a Parkinson’s and Alzheimer’s disease cellular model. Biomolecules. 2025;15(9):1322. doi: 10.3390/biom15091322. PMC12467174 41008629

[83] Wang X, Negrou E, Maloney MT, et al. Understanding LRRK2 kinase activity in preclinical models and human subjects through quantitative analysis of LRRK2 and pT73 Rab10. Sci Rep. 2021;11(1):12900. doi: 10.1038/s41598-021-91943-4. 34145320 PMC8213766

[84] Han B-S, Iacovitti L, Katano T, et al. Expression of the LRRK2 gene in the midbrain dopaminergic neurons of the substantia nigra. Neurosci Lett. 2008;442(3):190–194. doi: 10.1016/j.neulet.2008.06.086. 18634852 PMC2737127

[85] Galter D, Westerlund M, Carmine A, et al. LRRK2 expression linked to dopamine‐innervated areas. Ann Neurol. 2006;59(4):714–719. doi: 10.1002/ana.20808. 16532471

[86] Melrose H, Lincoln S, Tyndall G, et al. Anatomical localization of leucine-rich repeat kinase 2 in mouse brain. Neuroscience. 2006;139(3):791–794. doi: 10.1016/j.neuroscience.2006.01.017. 16504409

[87] Simón‐Sánchez J, Herranz‐Pérez V, Olucha‐Bordonau F, et al. LRRK2 is expressed in areas affected by Parkinson’s disease in the adult mouse brain. Eur J of Neuroscience. 2006;23(3):659–666. doi: 10.1111/j.1460-9568.2006.04616.x. 16487147

[88] Dzamko N, Gysbers AM, Bandopadhyay R, et al. LRRK2 levels and phosphorylation in Parkinson’s disease brain and cases with restricted Lewy bodies. Mov Disord. 2017;32(3):423–432. doi: 10.1002/mds.26892. 27911006

[89] Masotti B, Tombesi G, Parisiadou L, et al. LRRK2 and the fragile synapse: a molecular prelude to Parkinson’s disease? Biochem J. 2025;482(21):1585–1605. doi: 10.1042/BCJ20253351. 41148193 PMC12687443

[90] Tian Y, Lv J, Su Z, et al. LRRK2 plays essential roles in maintaining lung homeostasis and preventing the development of pulmonary fibrosis. Proc Natl Acad Sci USA. 2021;118(35):e2106685118. doi: 10.1073/pnas.2106685118. PMC8536316 34446559

[91] Looyenga BD, Furge KA, Dykema KJ, et al. Chromosomal amplification of leucine-rich repeat kinase-2 (LRRK2) is required for oncogenic MET signaling in papillary renal and thyroid carcinomas. Proc Natl Acad Sci USA. 2011;108(4):1439–1444. doi: 10.1073/pnas.1012500108. 21220347 PMC3029686

[92] Ahmadi Rastegar D, Dzamko N. Leucine rich repeat kinase 2 and innate immunity. Front Neurosci. 2020;14:193. doi: 10.3389/fnins.2020.00193. 32210756 PMC7077357

[93] Gardet A, Benita Y, Li C, et al. LRRK2 is involved in the IFN-γ response and host response to pathogens. J Immunol. 2010;185(9):5577–5585. doi: 10.4049/jimmunol.1000548. 20921534 PMC3156100

[94] Hakimi M, Selvanantham T, Swinton E, et al. Parkinson’s disease-linked LRRK2 is expressed in circulating and tissue immune cells and upregulated following recognition of microbial structures. J Neural Transm (Vienna). 2011;118(5):795–808. doi: 10.1007/s00702-011-0653-2. 21552986 PMC3376651

[95] Cook DA, Kannarkat GT, Cintron AF, et al. LRRK2 levels in immune cells are increased in Parkinson’s disease. NPJ Parkinsons Dis. 2017;3(1):11–11. doi: 10.1038/s41531-017-0010-8. 28649611 PMC5459798

[96] Thévenet J, Pescini Gobert R, Hooft van Huijsduijnen R, et al. Regulation of LRRK2 expression points to a functional role in human monocyte maturation. PLoS One. 2011;6(6):e21519-e21519. doi: 10.1371/journal.pone.0021519. 21738687 PMC3124520

[97] Wang J, Song W. Regulation of LRRK2 promoter activity and gene expression by Sp1. Mol Brain. 2016;9(1):33–33. doi: 10.1186/s13041-016-0215-5. 27004687 PMC4802577

[98] Zhao J, Molitor TP, Langston JW, et al. LRRK2 dephosphorylation increases its ubiquitination. Biochem J. 2015;469(1):107–120. doi: 10.1042/BJ20141305. 25939886 PMC4613513

[99] Deyaert E, Wauters L, Guaitoli G, et al. A homologue of the Parkinson’s disease-associated protein LRRK2 undergoes a monomer-dimer transition during GTP turnover. Nat Commun. 2017;8(1):1008–1008. doi: 10.1038/s41467-017-01103-4. 29044096 PMC5714945

[100] Gilsbach BK, et al. Intramolecular feedback regulation of the LRRK2 Roc G domain by a LRRK2 kinase dependent mechanism. 2023 10.7554/eLife.91083 PMC11658767 39699947

[101] Nixon-Abell J, Berwick DC, Grannó S, et al. Protective LRRK2 R1398H variant enhances GTPase and Wnt signaling activity. Front Mol Neurosci. 2016;9:18. doi: 10.3389/fnmol.2016.00018. 27013965 PMC4781896

[102] Xiong Y, Coombes CE, Kilaru A, et al. GTPase activity plays a key role in the pathobiology of LRRK2. PLoS Genet. 2010;6(4):e1000902–e1000902. doi: 10.1371/journal.pgen.1000902. 20386743 PMC2851569

[103] Healy DG, Falchi M, O’Sullivan SS, et al. Phenotype, genotype, and worldwide genetic penetrance of LRRK2-associated Parkinson’s disease: a case-control study. Lancet Neurol. 2008;7(7):583–590. doi: 10.1016/S1474-4422(08)70117-0. 18539534 PMC2832754

[104] Islam MS, Moore DJ. Mechanisms of LRRK2-dependent neurodegeneration: role of enzymatic activity and protein aggregation. Biochem Soc Trans. 2017;45(1):163–172. doi: 10.1042/BST20160264. 28202670 PMC5521802

[105] Daniëls V, Vancraenenbroeck R, Law BMH, et al. Insight into the mode of action of the LRRK2 Y1699C pathogenic mutant. J Neurochem. 2011;116(2):304–315. doi: 10.1111/j.1471-4159.2010.07105.x. 21073465 PMC3005098

[106] Tezuka T, Taniguchi D, Sano M, et al. Pathophysiological evaluation of the LRRK2 G2385R risk variant for Parkinson’s disease. NPJ Parkinsons Dis. 2022;8(1):97–97. doi: 10.1038/s41531-022-00367-y. 35931783 PMC9355991

[107] Rocha EM, Keeney MT, Di Maio R, et al. LRRK2 and idiopathic Parkinson’s disease. Trends Neurosci. 2022;45(3):224–236. doi: 10.1016/j.tins.2021.12.002. 34991886 PMC8854345

[108] Paglini G, Kunda P, Quiroga S, et al. Suppression of radixin and moesin alters growth cone morphology, motility, and process formation in primary cultured neurons. J Cell Biol. 1998;143(2):443–455. doi: 10.1083/jcb.143.2.443. 9786954 PMC2132841

[109] Jaleel M, Nichols RJ, Deak M, et al. LRRK2 phosphorylates moesin at threonine-558: characterization of how Parkinson’s disease mutants affect kinase activity. Biochem J. 2007;405(2):307–317. doi: 10.1042/BJ20070209. 17447891 PMC1904520

[110] Di Maio R, Hoffman EK, Rocha EM, et al. LRRK2 activation in idiopathic Parkinson’s disease. Sci Transl Med. 2018;10(451):eaar5429. doi: 10.1126/scitranslmed.aar5429. PMC6344941 30045977

[111] Daher JPL, Abdelmotilib HA, Hu X, et al. Leucine-rich repeat kinase 2 (LRRK2) pharmacological inhibition abates α-synuclein gene-induced neurodegeneration. J Biol Chem. 2015;290(32):19433–19444. doi: 10.1074/jbc.M115.660001. 26078453 PMC4528108

[112] Gersel Stokholm M, Garrido A, Tolosa E, et al. Imaging dopamine function and microglia in asymptomatic LRRK2 mutation carriers. J Neurol. 2020;267(8):2296–2300. doi: 10.1007/s00415-020-09830-3. 32318850 PMC7359140

[113] McManus RM, Heneka MT. Role of neuroinflammation in neurodegeneration: new insights. Alzheimers Res Ther. 2017;9(1):14–14. doi: 10.1186/s13195-017-0241-2. 28259169 PMC5336609

[114] Fuzzati-Armentero MT, Cerri S, Blandini F. Peripheral-central neuroimmune crosstalk in Parkinson’s disease: what do patients and animal models tell us? Front Neurol. 2019;10:232. doi: 10.3389/fneur.2019.00232. 30941089 PMC6433876

[115] Maiuolo J, Gliozzi M, Musolino V, et al. The “Frail” brain blood barrier in neurodegenerative diseases: role of early disruption of endothelial cell-to-cell connections. Int J Mol Sci. 2018;19(9):2693–2693. doi: 10.3390/ijms19092693. 30201915 PMC6164949

[116] Sommer A, Marxreiter F, Krach F, et al. Th17 lymphocytes induce neuronal cell death in a human iPSC-based model of Parkinson’s DISEASE. Cell Stem Cell. 2019;24(6):1006–1006. doi: 10.1016/j.stem.2019.04.019. 31173705

[117] Cabezudo D, Baekelandt V, Lobbestael E. Multiple-hit hypothesis in Parkinson’s disease: LRRK2 and inflammation. Front Neurosci. 2020;14:376. doi: 10.3389/fnins.2020.00376. 32410948 PMC7199384

[118] Wallings RL, Tansey MG. LRRK2 regulation of immune-pathways and inflammatory disease. Biochem Soc Trans. 2019;47(6):1581–1595. doi: 10.1042/BST20180463. 31769472 PMC6925522

[119] Li H-X, Zhang C, Zhang K, et al. Inflammatory bowel disease and risk of Parkinson’s disease: evidence from a meta-analysis of 14 studies involving more than 13.4 million individuals. Front Med (Lausanne). 2023;10:1137366. doi: 10.3389/fmed.2023.1137366. 37153103 PMC10157095

[120] Hirsch EC, Hunot S. Neuroinflammation in Parkinson’s disease: a target for neuroprotection? Lancet Neurol. 2009;8(4):382–397. doi: 10.1016/S1474-4422(09)70062-6. 19296921

[121] He Y, Le W-D, Appel SH. Role of Fcγ receptors in nigral cell injury induced by Parkinson disease immunoglobulin injection into mouse substantia Nigra. Exp Neurol. 2002;176(2):322–327. doi: 10.1006/exnr.2002.7946. 12359173

[122] Choi I, Kim B, Byun J-W, et al. LRRK2 G2019S mutation attenuates microglial motility by inhibiting focal adhesion kinase. Nat Commun. 2015;6(1):8255. doi: 10.1038/ncomms9255. 26365310 PMC4647842

[123] Moehle MS, Webber PJ, Tse T, et al. LRRK2 inhibition attenuates microglial inflammatory responses. J Neurosci. 2012;32(5):1602–1611. doi: 10.1523/JNEUROSCI.5601-11.2012. 22302802 PMC3532034

[124] Zheng Z, Zhang S, Liu X, et al. LRRK2 regulates ferroptosis through the system Xc‐GSH–GPX4 pathway in the neuroinflammatory mechanism of Parkinson’s disease. J Cell Physiol. 2024;239(5):e31250. doi: 10.1002/jcp.31250. 38477420

[125] Liu X, Zheng Z, Xue C, et al. LRRK2 mediates α-synuclein-induced neuroinflammation and ferroptosis through the p62-Keap1-Nrf2 pathway in Parkinson’s disease. Inflammation. 2025;48(5):3666–3691. doi: 10.1007/s10753-025-02291-8. 40169487 PMC12596413

[126] He K-J, Zhang J-B, Liu J-Y, et al. LRRK2 G2019S promotes astrocytic inflammation induced by oligomeric α-synuclein through NF-κB pathway. iScience. 2023;26(11):108130–108130. doi: 10.1016/j.isci.2023.108130. 37876795 PMC10590863

[127] Streubel-Gallasch L, et al. Parkinson’s disease–associated LRRK2 interferes with astrocyte-mediated alpha-synuclein clearance. Mol Neurobiol. 2021;58(7):3119–3140. doi: 10.1007/s12035-021-02327-8. 33629273 PMC8257537

[128] Galper J, Kim WS, Dzamko N. LRRK2 and lipid pathways: implications for Parkinson’s disease. Biomolecules. 2022;12(11):1597–1597. doi: 10.3390/biom12111597. 36358947 PMC9687231

[129] Maloney MT, et al. LRRK2 kinase activity regulates Parkinson’s disease-relevant lipids at the lysosome. 2022. 10.1186/s13024-025-00880-7 PMC12330094 40770658

[130] Ferrazza R, Cogo S, Melrose H, et al. LRRK2 deficiency impacts ceramide metabolism in brain. Biochem Biophys Res Commun. 2016;478(3):1141–1146. doi: 10.1016/j.bbrc.2016.08.082. 27539321

[131] Erb ML, Moore DJ. LRRK2 and the endolysosomal system in Parkinson’s disease. J Parkinsons Dis. 2020;10(4):1271–1291. doi: 10.3233/JPD-202138. 33044192 PMC7677880

[132] Piccoli G, Volta M. LRRK2 along the Golgi and lysosome connection: a jamming situation. Biochem Soc Trans. 2021;49(5):2063–2072. doi: 10.1042/BST20201146. 34495322 PMC8589420

[133] Esteves A, CardosoS. LRRK2 a pivotal player in mitochondrial dynamics and lysosomal clustering: highlights to sporadic Parkinson’s disease. Ther Targets Neurol Dis. 2015;2:e629. doi: 10.14800/ttnd.629.

[134] Esteves AR, G-Fernandes M, Santos D, et al. The Upshot of LRRK2 inhibition to Parkinson’s disease paradigm. Mol Neurobiol. 2015;52(3):1804–1820. doi: 10.1007/s12035-014-8980-6. 25394383

[135] Kedariti M, et al. The activities of LRRK2 and GCase are positively correlated in clinical biospecimens and experimental models of Parkinson’s disease. 2021.

[136] Hsieh C-H, Shaltouki A, Gonzalez AE, et al. Functional impairment in miro degradation and mitophagy is a shared feature in familial and sporadic Parkinson’s disease. Cell Stem Cell. 2016;19(6):709–724. doi: 10.1016/j.stem.2016.08.002. 27618216 PMC5135570

[137] Orenstein SJ, Kuo S-H, Tasset I, et al. Interplay of LRRK2 with chaperone-mediated autophagy. Nat Neurosci. 2013;16(4):394–406. doi: 10.1038/nn.3350. 23455607 PMC3609872

[138] Buck SA, Sanders LH. LRRK2-mediated mitochondrial dysfunction in Parkinson’s disease. Biochem J. 2025;482(11):721–739. doi: 10.1042/BCJ20253062. 40440058 PMC12181902

[139] Esteves AR, Arduíno DM, Silva DFF, et al. Mitochondrial dysfunction: the road to alpha-synuclein oligomerization in PD. Parkinsons Dis. 2011;2011:693761–693720. doi: 10.4061/2011/693761. 21318163 PMC3026982

[140] Pereira C, Miguel Martins L, Saraiva L. LRRK2, but not pathogenic mutants, protects against H2O2 stress depending on mitochondrial function and endocytosis in a yeast model. Biochim Biophys Acta. 2014;1840(6):2025–2031. doi: 10.1016/j.bbagen.2014.02.015. 24576675

[141] Heo HY, Park J-M, Kim C-H, et al. LRRK2 enhances oxidative stress-induced neurotoxicity via its kinase activity. Exp Cell Res. 2010;316(4):649–656. doi: 10.1016/j.yexcr.2009.09.014. 19769964

[142] Liou AKF, Leak RK, Li L, et al. Wild-type LRRK2 but not its mutant attenuates stress-induced cell death via ERK pathway. Neurobiol Dis. 2008;32(1):116–124. doi: 10.1016/j.nbd.2008.06.016. 18675914 PMC2580823

[143] Long S, Guo W, Hu S, et al. G2019S LRRK2 increases stress susceptibility through inhibition of DAF-16 nuclear translocation in a 14-3-3 associated-manner in caenorhabditis elegans. Front Neurosci. 2018;12:782. doi: 10.3389/fnins.2018.00782. 30464741 PMC6234837

[144] Loeffler DA, Klaver AC, Coffey MP, et al. Increased oxidative stress markers in cerebrospinal fluid from healthy subjects with Parkinson’s disease-associated LRRK2 gene mutations. Front Aging Neurosci. 2017;9:89. doi: 10.3389/fnagi.2017.00089. 28420983 PMC5376564

[145] Mendivil-Perez M, Velez-Pardo C, Jimenez-Del-Rio M. Neuroprotective effect of the LRRK2 kinase inhibitor PF-06447475 in human nerve-like differentiated cells exposed to oxidative stress stimuli: implications for Parkinson’s disease. Neurochem Res. 2016;41(10):2675–2692. doi: 10.1007/s11064-016-1982-1. 27394417

[146] Quintero-Espinosa DA, Sanchez-Hernandez S, Velez-Pardo C, et al. LRRK2 KNOCKOUT CONFERS RESISTance in HEK-293 cells to rotenone-induced oxidative stress, mitochondrial damage, and apoptosis. Int J Mol Sci. 2023;24(13):10474–10474. doi: 10.3390/ijms241310474. 37445652 PMC10341561

[147] Kawakami F, Imai M, Tamaki S, et al. Nrf2 expression is decreased in LRRK2 transgenic mouse brain and LRRK2 overexpressing SH-SY5Y cells. Biol Pharm Bull. 2023;46(1):123–127. doi: 10.1248/bpb.b22-00356. 36596520

[148] Keeney MT, Rocha EM, Hoffman EK, et al. LRRK2 regulates production of reactive oxygen species in cell and animal models of Parkinson’s disease. Sci Transl Med. 2024;16(767):eadl3438. doi: 10.1126/scitranslmed.adl3438. 39356746 PMC13280971

[149] Rocha EM, De Miranda BR, Castro S, et al. LRRK2 inhibition prevents endolysosomal deficits seen in human Parkinson’s disease. Neurobiol Dis. 2020;134:104626–104626. doi: 10.1016/j.nbd.2019.104626. 31618685 PMC7345850

[150] Hattula K, Furuhjelm J, Tikkanen J, et al. Characterization of the Rab8-specific membrane traffic route linked to protrusion formation. J Cell Sci. 2006;119(Pt 23):4866–4877. doi: 10.1242/jcs.03275. 17105768

[151] Rivero-Ríos P, Romo-Lozano M, Madero-Pérez J, et al. The G2019S variant of leucine-rich repeat kinase 2 (LRRK2) alters endolysosomal trafficking by impairing the function of the GTPase RAB8A. J Biol Chem. 2019;294(13):4738–4758. doi: 10.1074/jbc.RA118.005008. 30709905 PMC6442034

[152] Mamais A, Kluss JH, Bonet-Ponce L, et al. Mutations in LRRK2 linked to Parkinson disease sequester Rab8a to damaged lysosomes and regulate transferrin-mediated iron uptake in microglia. PLoS Biol. 2021;19(12):e3001480. doi: 10.1371/journal.pbio.3001480. 34914695 PMC8675653

[153] Jia R, Liu Y, Shuai K, et al. The relationship between iron and LRRK2 in a 6-OHDA-Induced Parkinson’s disease model. Int J Mol Sci. 2023;24(4):3709–3709. doi: 10.3390/ijms24043709. 36835121 PMC9964371

[154] Schapansky J, Khasnavis S, DeAndrade MP, et al. Familial knockin mutation of LRRK2 causes lysosomal dysfunction and accumulation of endogenous insoluble α-synuclein in neurons. Neurobiol Dis. 2018;111:26–35. doi: 10.1016/j.nbd.2017.12.005. 29246723 PMC5803451

[155] Xiong Y, Dawson TM, Dawson VL. Models of LRRK2-associated Parkinson’s disease. Adv Neurobiol. 2017;14:163–191. doi: 10.1007/978-3-319-49969-7_9. 28353284 PMC5535810

[156] Blauwendraat C, Reed X, Kia DA, et al. Frequency of loss of function variants in LRRK2 in Parkinson disease. JAMA Neurol. 2018;75(11):1416–1422. doi: 10.1001/jamaneurol.2018.1885. 30039155 PMC6248108

[157] Whiffin N, Armean IM, Kleinman A, et al. The effect of LRRK2 loss-of-function variants in humans. Nat Med. 2020;26(6):869–877. doi: 10.1038/s41591-020-0893-5. 32461697 PMC7303015

[158] Jennings D, Huntwork-Rodriguez S, Henry AG, et al. Preclinical and clinical evaluation of the LRRK2 inhibitor DNL201 for Parkinson’s disease. Sci Transl Med. 2022;14(648):eabj2658. doi: 10.1126/scitranslmed.abj2658. 35675433

[159] Scott JD, DeMong DE, Greshock TJ, et al. Discovery of a 3-(4-Pyrimidinyl) Indazole (MLi-2), an orally available and selective leucine-rich repeat kinase 2 (LRRK2) inhibitor that reduces brain kinase activity. J Med Chem. 2017;60(7):2983–2992. doi: 10.1021/acs.jmedchem.7b00045. 28245354

[160] Lesniak RK, Nichols RJ, Montine TJ. Development of mutation-selective LRRK2 kinase inhibitors as precision medicine for Parkinson’s disease and other diseases for which carriers are at increased risk. Front Neurol. 2022;13:1016040. doi: 10.3389/fneur.2022.1016040. 36388213 PMC9643380

[161] Alcalay RN, Hsieh F, Tengstrand E, et al. Higher urine bis(Monoacylglycerol)phosphate levels in LRRK2 G2019S mutation carriers: implications for therapeutic development. Mov Disord. 2020;35(1):134–141. doi: 10.1002/mds.27818. 31505072 PMC6981003

[162] Meneses-Salas E, Castellá M, Arnold M, et al. Extracellular vesicle-mediated release of bis(monoacylglycerol)phosphate is regulated by LRRK2 and Glucocerebrosidase activity. Elife. 2026;14:RP106330. doi: 10.7554/eLife.106330. PMC13046380 41925724

[163] Fuji RN, Flagella M, Baca M, et al. Effect of selective LRRK2 kinase inhibition on nonhuman primate lung. Sci Transl Med. 2015;7(273):273ra15. doi: 10.1126/scitranslmed.aaa3634. 25653221

[164] Jennings D, Huntwork-Rodriguez S, Vissers MFJM, et al. LRRK2 inhibition by BIIB122 in healthy participants and patients with Parkinson’s disease. Mov Disord. 2023;38(3):386–398. doi: 10.1002/mds.29297. 36807624

[165] Gomes S, Garrido A, Tonelli F, et al. Elevated urine BMP phospholipids in LRRK2 and VPS35 mutation carriers with and without Parkinson’s disease. NPJ Parkinsons Dis. 2023;9(1):52. doi: 10.1038/s41531-023-00482-4. 37015928 PMC10073226

[166] Fraser KB, Rawlins AB, Clark RG, et al. Ser(P)‐1292 LRRK2 in urinary exosomes is elevated in idiopathic Parkinson’s disease. Mov Disord. 2016;31(10):1543–1550. doi: 10.1002/mds.26686. 27297049 PMC5053851

[167] Herzig MC, Kolly C, Persohn E, et al. LRRK2 protein levels are determined by kinase function and are crucial for kidney and lung homeostasis in mice. Hum Mol Genet. 2011;20(21):4209–4223. doi: 10.1093/hmg/ddr348. 21828077 PMC3188995

[168] Hu J, Zhang D, Tian K, et al. Small-molecule LRRK2 inhibitors for PD therapy: current achievements and future perspectives. Eur J Med Chem. 2023;256:115475–115475. doi: 10.1016/j.ejmech.2023.115475. 37201428

[169] Gillardon F, Kremmer E, Froehlich T, et al. ATP-competitive LRRK2 inhibitors interfere with monoclonal antibody binding to the kinase domain of LRRK2 under native conditions. A method to directly monitor the active conformation of LRRK2? J Neurosci Methods. 2013;214(1):62–68. doi: 10.1016/j.jneumeth.2012.12.015. 23318290

[170] Schaffner A, Li X, Gomez-Llorente Y, et al. Vitamin B12 modulates Parkinson’s disease LRRK2 kinase activity through allosteric regulation and confers neuroprotection. Cell Res. 2019;29(4):313–329. doi: 10.1038/s41422-019-0153-8. 30858560 PMC6462009

[171] Häbig K, Walter M, Poths S, et al. RNA interference of LRRK2-microarray expression analysis of a Parkinson’s disease key player. Neurogenetics. 2008;9(2):83–94. doi: 10.1007/s10048-007-0114-0. 18097693

[172] Wu Q, Xi DZ, Wang YH. MicroRNA-599 regulates the development of Parkinson’s disease through mediating LRRK2 expression. Eur Rev Med Pharmacol Sci. 2019;23(2):724–731. doi: 10.26355/eurrev_201901_16886. 30720180

[173] Zhao HT, John N, Delic V, et al. LRRK2 antisense oligonucleotides ameliorate α-synuclein inclusion formation in a Parkinson’s disease mouse model. Mol Ther Nucleic Acids. 2017;8:508–519. doi: 10.1016/j.omtn.2017.08.002. 28918051 PMC5573879

[174] Vermilyea SC, Babinski A, Tran N, et al. In vitro CRISPR/Cas9-directed gene editing to model LRRK2 G2019S Parkinson’s disease in common marmosets. Sci Rep. 2020;10(1):3447. doi: 10.1038/s41598-020-60273-2. 32103062 PMC7044232

[175] Lee S-Y, Chung S-K. Generation of gene-corrected iPSC line, KIOMi002-A, from Parkinson’s disease patient iPSC with LRRK2 G2019S mutation using BAC-based homologous recombination. Stem Cell Res. 2019;41:101649–101649. doi: 10.1016/j.scr.2019.101649. 31731184

[176] Tomkins JE, Dihanich S, Beilina A, et al. Comparative protein interaction network analysis identifies shared and distinct functions for the human ROCO proteins. Proteomics. 2018;18(10):e1700444. doi: 10.1002/pmic.201700444. 29513927 PMC5992104

[177] Pérez-Carrión MD, Posadas I, Solera J, et al. LRRK2 and proteostasis in Parkinson’s disease. Int J Mol Sci. 2022;23(12):6808–6808. doi: 10.3390/ijms23126808. 35743250 PMC9224256

[178] Azeggagh S, Berwick DC. The development of inhibitors of leucine‐rich repeat kinase 2 (LRRK2) as a therapeutic strategy for Parkinson’s disease: the current state of play. Br J Pharmacol. 2022;179(8):1478–1495. doi: 10.1111/bph.15575. 34050929

[179] Singh RK, Soliman A, Guaitoli G, et al. Nanobodies as allosteric modulators of Parkinson’s disease–associated LRRK2. Proc Natl Acad Sci USA. 2022;119(9):e2112712119. doi: 10.1073/pnas.2112712119. PMC8892280 35217606

[180] Dusonchet J, Li H, Guillily M, et al. A Parkinson’s disease gene regulatory network identifies the signaling protein RGS2 as a modulator of LRRK2 activity and neuronal toxicity. Hum Mol Genet. 2014;23(18):4887–4905. doi: 10.1093/hmg/ddu202. 24794857 PMC4140468

[181] Stafa K, Trancikova A, Webber PJ, et al. GTPase activity and neuronal toxicity of Parkinson’s disease–associated LRRK2 is regulated by ArfGAP1. PLoS Genet. 2012;8(2):e1002526-e1002526. doi: 10.1371/journal.pgen.1002526. 22363216 PMC3280333

[182] Melachroinou K, Leandrou E, Valkimadi P-E, et al. Activation of FADD-dependent neuronal death pathways as a predictor of pathogenicity for LRRK2 mutations. PLoS One. 2016;11(11):e0166053-e0166053. doi: 10.1371/journal.pone.0166053. 27832104 PMC5104429

[183] Xiromerisiou G, Hadjigeorgiou GM, Gourbali V, et al. Screening for SNCA and LRRK2 mutations in Greek sporadic and autosomal dominant Parkinson’s disease: identification of two novel LRRK2 variants. Eur J Neurol. 2007;14(1):7–11. doi: 10.1111/j.1468-1331.2006.01551.x. 17222106

[184] Coku I, Mutez E, Eddarkaoui S, et al. Functional analyses of two novel LRRK2 pathogenic variants in familial Parkinson’s disease. Mov Disord. 2022;37(8):1761–1767. doi: 10.1002/mds.29124. 35708213 PMC9543145

[185] Kishore A, Ashok Kumar Sreelatha A, Sturm M, et al. Understanding the role of genetic variability in LRRK2 in Indian population. Mov Disord. 2019;34(4):496–505. doi: 10.1002/mds.27558. 30485545 PMC8985845

[186] Ross OA, Soto-Ortolaza AI, Heckman MG, et al. Association of LRRK2 exonic variants with susceptibility to Parkinson’s disease: a case–control study. Lancet Neurol. 2011;10(10):898–908. doi: 10.1016/S1474-4422(11)70175-2. 21885347 PMC3208320

[187] Kalogeropulou AF, Purlyte E, Tonelli F, et al. Impact of 100 LRRK2 variants linked to Parkinson’s disease on kinase activity and microtubule binding. Biochem J. 2022;479(17):1759–1783. doi: 10.1042/BCJ20220161. 35950872 PMC9472821

[188] Zhang P, Fan Y, Ru H, et al. Crystal structure of the WD40 domain dimer of LRRK2. Proc Natl Acad Sci USA. 2019;116(5):1579–1584. doi: 10.1073/pnas.1817889116. 30635421 PMC6358694

[189] Li T, Ning B, Kong L, et al. A LRRK2 GTP binding inhibitor, 68, reduces LPS-induced signaling events and TNF-alpha release in human lymphoblasts. Cells. 2021;10(2):480. doi: 10.3390/cells10020480. PMC7926966 33672296

[190] Nguyen AP, Moore DJ. Understanding the GTPase activity of LRRK2: regulation, function, and neurotoxicity. Adv Neurobiol. 2017;14:71–88. doi: 10.1007/978-3-319-49969-7_4. 28353279 PMC5521808

